# Deciphering ferroptosis in critical care: mechanisms, consequences, and therapeutic opportunities

**DOI:** 10.3389/fimmu.2024.1511015

**Published:** 2024-12-16

**Authors:** Ruimin Tan, Chen Ge, Yating Yan, He Guo, Xumin Han, Qiong Zhu, Quansheng Du

**Affiliations:** ^1^ School of Clinical Medical, North China University of Science and Technology, Tangshan, Hebei, China; ^2^ Critical Care Department, Hebei General Hospital, Shijiazhuang, Hebei, China; ^3^ School of Graduate, Hebei Medical University, Shijiazhuang, Hebei, China; ^4^ Department of Orthopaedics, The People’s Hospital Of Shizhu, Chongqing, China

**Keywords:** ferroptosis, critical illness, iron overload, lipid metabolism, mitochondrial dysfunction

## Abstract

Ischemia-reperfusion injuries (IRI) across various organs and tissues, along with sepsis, significantly contribute to the progression of critical illnesses. These conditions disrupt the balance of inflammatory mediators and signaling pathways, resulting in impaired physiological functions in human tissues and organs. Ferroptosis, a distinct form of programmed cell death, plays a pivotal role in regulating tissue damage and modulating inflammatory responses, thereby influencing the onset and progression of severe illnesses. Recent studies highlight that pharmacological agents targeting ferroptosis-related proteins can effectively mitigate oxidative stress caused by IRI in multiple organs, alleviating associated symptoms. This manuscript delves into the mechanisms and signaling pathways underlying ferroptosis, its role in critical illnesses, and its therapeutic potential in mitigating disease progression. We aim to offer a novel perspective for advancing clinical treatments for critical illnesses.

## 1 Introduction

Critical illness, marked by organ-level pathophysiological disruptions, remains a significant global health challenge (1, 2). Despite its impact, intensive care unit (ICU) outcomes are often underrepresented in global disease burden studies. For instance, a 2017 report revealed that sepsis-related deaths accounted for 47% of all fatalities worldwide, underscoring an urgent need for focused research (2, 3). While advancements in critical care have improved symptomatic management—such as organ support and fluid resuscitation—progress in targeted therapies has been limited. Identifying novel mechanisms and therapeutic strategies is, therefore, paramount. Mechanisms linked to metabolic dysregulation, including ferroptosis, autophagy, and oxidative stress—processes driven by lipid peroxidation and cellular ion imbalances—present promising avenues for innovation in critical care.

Cell death is a fundamental process critical for growth, homeostasis, and the progression of various diseases (2, 4). Programmed cell death pathways, such as necrosis, autophagy, pyroptosis, and apoptosis, operate through well-defined signaling regulatory mechanisms and are closely tied to disease pathophysiology (2, 5, 6). Among these, ferroptosis—an iron-dependent form of regulated cell death triggered by lipid peroxidation—has emerged as a key contributor to critical illnesses, including sepsis, acute respiratory distress syndrome (ARDS), acute kidney injury (AKI), and Ischemia-reperfusion injuries (IRI) (2, 7–9). Growing evidence underscores the importance of targeting ferroptosis to better understand and manage these life-threatening conditions. Iron, an essential trace element in the human body, is involved in numerous biological processes, including energy metabolism and nucleotide synthesis and repair (2, 10). While the concept of ferroptosis dates back to the 1980s, it was formally named by Dixon in 2012 (2, 7, 11). Ferroptosis development is driven by iron-induced reactive oxygen species (ROS), making it susceptible to inhibition by lipophilic antioxidants and agents such as ferritin and iron chelators (2, 12).

**Figure 1:**
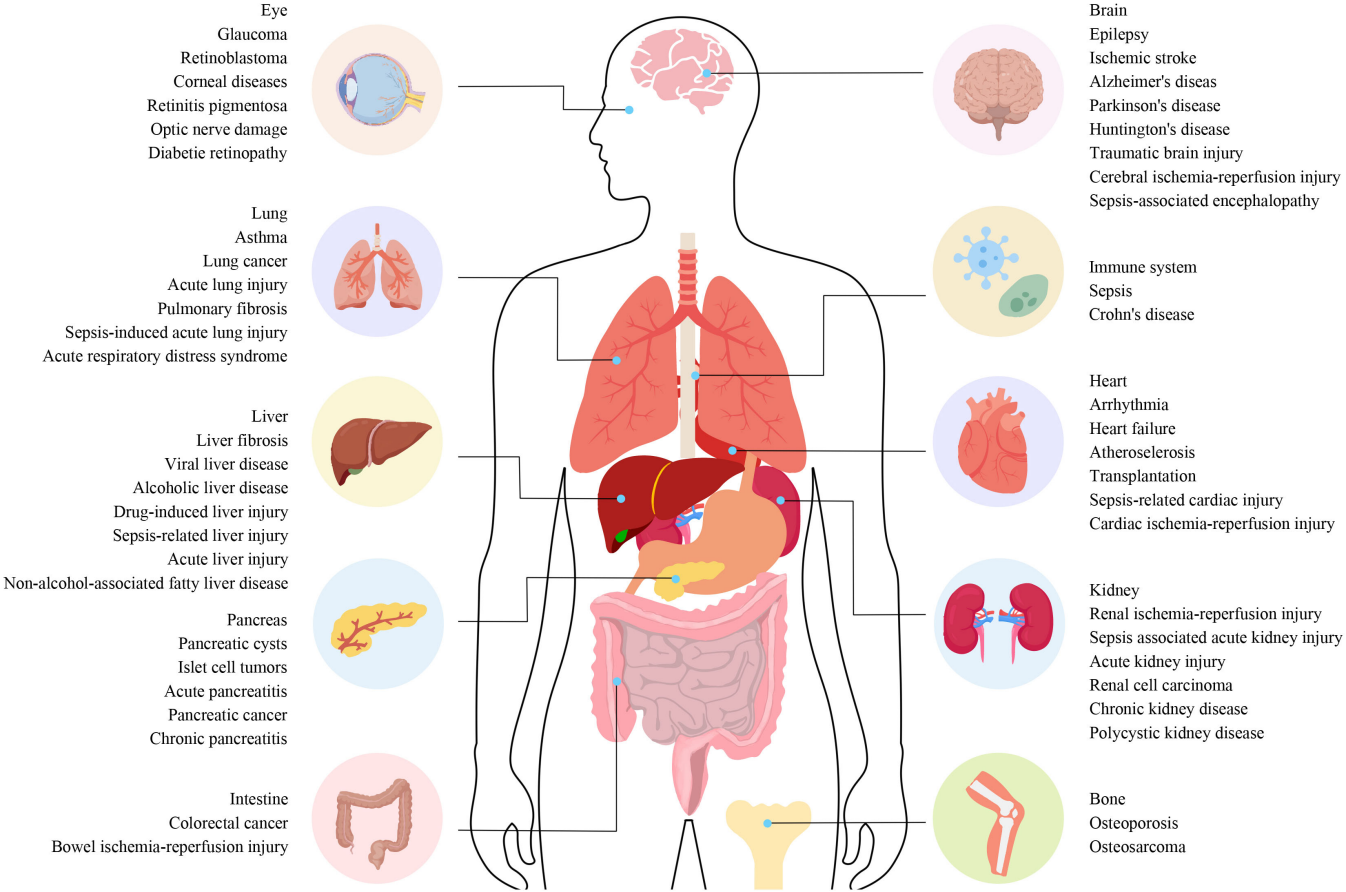
The connection between different diseases and ferroptosis. The connection between different diseases and ferroptosis. Ferroptosis plays a role in the regulation of various systemic diseases, such as diseases of the nervous system, cardiovascular system, digestive system, musculoskeletal system, autoimmune system, visual system, lung, liver, and kidney. Ferroptosis often involves systemic interactions, where iron metabolism and oxidative stress affect multiple organ systems. For example, diseases like sepsis can trigger widespread inflammation and organ failure involving ferroptotic pathways in the liver, lungs, kidneys, and cardiovascular system. Understanding these pathways is essential for developing targeted therapies that can mitigate ferroptosis-induced damage across various diseases.

Current evidence highlights ferroptosis as a key player in the onset and progression of various diseases, positioning it as a potential target for clinical therapies (**Figure 1**). This review summarizes the occurrence, characteristics, regulatory mechanisms, and critical molecular pathways of ferroptosis, emphasizing its direct and indirect roles in the etiology of serious disorders. Additionally, we explore potential therapeutic targets linked to ferroptosis, offering new insights for clinical applications in treating critical conditions.

## 2 Discovery of ferroptosis

In 1980, System Xc⁻ was identified as a transporter that exchanges glutamate for cystine, enabling cystine entry into cells (2, 13). In 2003, Dolma et al. discovered that NSC146109 (later known as erastin) selectively killed BJeLR cells with mutated Ras oncogenes during high-throughput screening (2, 14). In 2008, compounds like RAS-selective lethal small molecules 3 (RSL3) and RSL5 were identified as non-apoptotic agents that induce cell death (2, 15). This form of cell death is distinct from previously recognized categories, as apoptosis, necrosis, and autophagy inhibitors did not prevent cell death induced by these compounds. However, significant inhibition could be achieved using iron chelators and lipid peroxide inhibitors (2, 16). In 2012, Dixon et al. demonstrated that erastin, a product linked to the Ras oncogene, induced cell death in tumor cells and coined the term ferroptosis for this iron-dependent mode of cell death (2, 11). The concept of ferroptosis gained formal recognition in 2018 when the Cell Death Nomenclature Committee defined it as a potentially regulatory mode of cell death (2, 17).

## 3 Ferroptosis and other types of cell death

Ferroptosis differs from other forms of programmed cell death by exhibiting distinct morphological features (2, 18). These include a reduction or absence of mitochondrial cristae, rupture of the outer mitochondrial membrane, mitochondrial shrinkage, and an increase in membrane density. While nuclear size remains normal, chromatin condensation is absent, and membrane blebbing occurs without full rupture (2, 19, 20). Biochemically, ferroptosis is marked by elevated iron and ROS levels, decreased glutathione (GSH), and reduced glutathione peroxidase 4 (GPX4) activity. It also involves disruption of the cysteine uptake system, changes in mitochondrial membrane potential, and arachidonic acid-mediated release of functional factors, all leading to lipid peroxidation and mitochondrial dysfunction. Additionally, specific gene expression changes have been observed, and ongoing studies are exploring the relationship between ferroptosis and other forms of cell death (21).

### 3.1 Ferroptosis and apoptosis

Both ferroptosis and apoptosis are programmed cell death mechanisms, but they differ in their biological characteristics, triggers, and signaling pathways, though there are notable intersections (22). For instance, B-cell lymphoma-2 (Bcl-2) family proteins, key regulators in the mitochondrial pathway of apoptosis, also influence ferroptosis. These proteins interact with Beclin1 (BECN1) to inhibit autophagy and ferroptosis, prevent cytochrome c release from mitochondria in apoptosis to block cell death, and regulate lipid metabolism and redox status in ferroptosis to prevent its occurrence (23, 24). Additionally, in cancer therapy, inducing ferroptosis is considered a strategy to target apoptotic cancer cells, particularly those resistant to conventional apoptosis-inducing treatments.

### 3.2 Ferroptosis and necroptosis

In some cases, the signaling pathways of ferroptosis and necroptosis converge. For example, under certain stress conditions, receptor-interacting protein kinase 1 (RIPK1) not only mediates necroptosis but can also influence ferroptosis by modulating cellular redox status (25). RIPK1 regulates the activity of antioxidant enzymes, indirectly affecting lipid peroxide levels and either promoting or inhibiting ferroptosis (26). During ferroptosis, cell membrane rupture releases several damage-associated molecular patterns (DAMPs), such as high-mobility group box 1 (HMGB1), which activate immune cells and trigger inflammation. Necroptosis, an inflammatory mode of cell death, similarly leads to membrane disruption through mixed lineage kinase domain-like protein (MLKL), causing the release of numerous inflammatory factors (27). Inflammation-related diseases may involve both ferroptosis and necroptosis, acting synergistically to exacerbate tissue damage and inflammation.

### 3.3 Ferroptosis and necrotic death

The transition from necrotic death to ferroptosis can be facilitated by phosphatidylethanolamine binding protein 1 (PEBP-1) and 15-lipoxygenase (28). Belavgeni et al. suggest that necrotic death may represent an initial phase in the ferroptosis-mediated spread of cell death (29). A small-molecule inhibitor, necrostatin-1f, was developed to investigate this relationship. Although it effectively inhibits necrotic death, it weakly inhibits ferroptosis, highlighting the distinct yet interconnected nature of these cell death pathways (30).

### 3.4 Ferroptosis and pyroptosis

Pyroptosis can be induced by ROS-generating drugs and iron ions through the ROS-Tom20-Caspase3-GSDME signaling pathway (31). During ferroptosis, lipid peroxides break down into 4-hydroxynonenal (4-HNE) and malondialdehyde (MDA), which interact with proteins, nucleophiles, and DNA bases, causing significant cytotoxicity (32). This process amplifies ROS signaling, activating mitochondrial caspase pathways typically associated with pyroptosis. These findings suggest a potential link between ferroptosis and pyroptosis, indicating an overlap between these cell death mechanisms.

### 3.5 Ferroptosis and autophagy

Ferroptosis regulation and progression are tightly linked to selective autophagy, which facilitates the degradation of key ferroptosis-related molecules and organelles. Recent studies highlight autophagy’s role in controlling ferritin levels, the primary iron storage protein (7). Nuclear receptor coactivator 4 (NCOA4), a transport receptor that selectively degrades ferritin, is a key player in regulating intracellular iron homeostasis. Overexpression of NCOA4 enhances ferritin degradation, increasing labile iron levels, which promotes ferroptosis (33).

### 3.6 Ferroptosis and oxidative death

First described in 2001, oxidative death is a non-apoptotic form of cell death characterized by glutathione depletion and oxidative stress (34). This process shares similarities with ferroptosis, as both activate the expression of eIF2α (35). Moreover, studies show that CRISPR/Cas9-mediated gene knockdown can protect synapses from both ferroptosis and oxidative death (36).

### 3.7 Ferroptosis and cuproptosis

Cuproptosis, a recently identified form of metal ion-dependent cell death, is driven by intracellular copper levels. Excess copper directly binds to fatty acylated components of the tricarboxylic acid cycle (TCA), disrupting protein homeostasis and causing the accumulation of fatty acylation-related proteins, ultimately triggering cell death (37). Recent studies suggest that ferroptosis inducers can initiate and accelerate cuproptosis in liver cancer. The concurrent use of ferroptosis and cuproptosis inducers leads to enhanced cell death (38).

### 3.8 Ferroptosis and disulfidptosis

Cancer cells with high levels of solute carrier family 7 member 11 (SLC7A11) undergo disulfidptosis, a thiol-dependent cell death induced by disulfide stress. This process is marked by the formation of multiple disulfide bonds in the cytoplasm, particularly under glucose starvation. This emerging phenomenon suggests a potential link between disulfidptosis and ferroptosis (39). However, evidence on the interaction between these two processes remains limited, and future research is needed to explore and clarify their relationship.

## 4 Regulatory mechanisms of ferroptosis

### 4.1 Regulation of iron homeostasis

Iron is essential for various physiological functions, and its homeostasis is crucial to preventing ferroptosis (40). Iron metabolism, which includes absorption, activation, storage, and recycling, is tightly regulated to ensure cellular health. Iron primarily enters the bloodstream through dietary intake and macrophage-mediated erythrocyte breakdown. It is absorbed in the Fe^3+^ form, binds to transferrin (TF), and is recognized by transferrin receptor 1 (TfR1) on cell membranes, after which the complex is internalized through endocytosis (41). TfR1 has been suggested as a potential marker for ferroptosis (42).

Inside cells, ferric reductase six-transmembrane epithelial antigen of prostate 3 (STEAP3) reduces Fe^3+^ to Fe^2+^, which binds to ferritin or is transported by ferroportin (Fpn) out of the cell. Divalent metal transporter 1 (DMT1) imports free Fe^2+^ from nucleosomes into the cytoplasm, contributing to the labile iron pool (LIP), where it plays a role in metabolic processes (43–45). Ferroportin and enzymes such as ceruloplasmin (CP) export Fe^2+^ from cells, converting it back to Fe^3+^ to maintain the iron cycle (46).

Iron homeostasis is tightly regulated by iron response elements (IREs) and iron regulatory proteins (IRPs), which control the translation of genes such as DMT1 and TfR1. Iron-regulated protein 2 (IRP2) binds to IREs under low-iron conditions to regulate iron levels, while IRP1 acts as a coenzyme when bound to an Fe-S cluster. When iron levels are high, IRP2 is degraded, and IRP1 remains bound to the Fe-S cluster, inhibiting further regulation and stabilizing iron homeostasis (47, 48).

The hepcidin-ferroportin-1 (Fpn1) axis is pivotal for iron regulation (49). Ferroportin exports iron, while ferritin stores excess iron (41, 50). In ferroportin-deficient mice, iron homeostasis is severely disrupted, leading to iron overload (51). Excessive iron, primarily stored in ferritin or as free Fe^2+^, can induce ferroptosis. Iron can overwhelm TF binding capacity, resulting in non-transferrin bound iron (NTBI), which catalyzes harmful reactions, such as Fenton and Haber-Weiss, producing ROS (52). These ROS damage lipids, driving ferroptosis. The transcription factor nuclear factor erythroid 2-related factor 2 (Nrf2), a leucine zipper protein, plays a crucial role in reducing excess iron and preventing ferroptosis by regulating iron intake, storage, and recycling.

In IRI, tissue damage occurs due to hypoxia during ischemia, and subsequent reperfusion further exacerbates ROS generation. Iron participates in the Fenton reaction, catalyzing the conversion of hydrogen peroxide into free radicals, thus intensifying cellular oxidative damage and potentially triggering ferroptosis. In sepsis, the inflammatory response significantly alters iron metabolism, redistributing iron from storage sites and decreasing serum iron levels. Excessive iron accumulation in cells and the reticuloendothelial system promotes ROS generation, induces ferroptosis, and exacerbates organ dysfunction. Inflammatory mediators in septic patients also regulate iron metabolism-related proteins, such as increasing hepcidin expression to drive iron accumulation. This retention and overload activate oxidative stress responses, damaging cellular structures and leading to tissue damage and organ failure.

### 4.2 Lipid metabolism

Ferroptosis is strongly influenced by lipid peroxidation, especially through polyunsaturated fatty acids (PUFAs), which undergo peroxidation in the presence of lipoxygenases and ROS. As integral components of cell membranes, PUFAs play key roles in immune response, inflammation, and cell proliferation (50). Free PUFAs, acting as precursors for lipid signals, must first esterify into membrane phospholipids and undergo oxidation to transmit ferroptosis signals. This process is facilitated by lysophosphatidylcholine acyltransferase 3 (LPCAT3) and acyl-CoA synthetase long-chain family member 4 (ACSL4) (53, 54).

Adrenoic acid (AdA) and arachidonic acid (AA) are primary PUFAs involved in inducing ferroptosis (54). ACSL4, in association with Coenzyme A (CoA), forms AA-CoA or AdA-CoA intermediates, which LPCAT3 esterifies into phosphatidylethanolamines, such as PE-AA or PE-AdA. These phospholipids are oxidized by lipid oxidases (LOXs) or via autoxidation, leading to cell death (53). ACSL4 also upregulates ferroptosis in a feedback loop, particularly when the neurofibromin 2-yes-associated protein 1 (NF2-YAP) pathway is inhibited (55).

Phosphorylation of ACSL4 enhances lipid peroxidation and facilitates the incorporation of PUFAs into plasmalogens, a process mediated by protein kinase C beta type isoform 2 (PKCβII) (55). In contrast, integrin α6β4 inhibits ferroptosis by downregulating ACSL4 expression. Moreover, thiazolidinedione hypoglycemic agents and safranin effectively suppress ferroptosis through ACSL4 targeting (56, 57). Knockdown of LPCAT3 confers protection against ferroptosis, underscoring its critical role (54, 57).

LOXs catalyze the oxidation of PUFAs both directly and within biofilms (53, 58). Among them, 15-LOX is particularly involved in lipid peroxidation, with its interaction with PUFAs, such as sn2-15-hydroperoxy-eicosatetraenoyl-phosphatidylethanolamines (sn2-15-HpETE-PE), playing a pivotal role in signaling ferroptosis (59). The involvement of 12-LOX in p53-mediated ferroptosis remains debated, despite its acknowledged importance (60).

Lipophagy, the autophagic degradation of lipid droplets (LDs), regulates ferroptosis by modulating lipid peroxidation (61). Inhibiting RAB7A or promoting tumor protein D52 (TPD52)-mediated lipid storage can prevent ferroptosis (61, 62). A deeper understanding of lipid peroxidation and its regulatory enzymes opens new therapeutic avenues for diseases such as cancer.

During the ischemic phase of IRI, oxygen and nutrient supply to cells is insufficient, disrupting energy metabolism and lipid homeostasis. Reperfusion leads to the influx of oxygen and iron ions, triggering oxidative reactions. Unsaturated fatty acids, particularly those in phospholipids containing polyunsaturated fatty acids, are prone to oxidation and serve as primary substrates for lipid peroxidation (62). Additionally, IRI affects the expression and activity of enzymes involved in lipid metabolism, altering intracellular lipid composition and further disrupting cellular homeostasis.

Sepsis accelerates the mobilization and oxidation of fatty acids, leading to an accumulation of lipid peroxidation byproducts such as 4-HNE and MDA. These products are toxic to cells, compromising membrane integrity and inducing cell death through ferroptosis (63). The inflammatory response during sepsis promotes fatty acid mobilization and increases the proportion of polyunsaturated fatty acids in cell membranes, heightening susceptibility to lipid peroxidation. This is particularly detrimental in endothelial cells and leukocytes, where lipid peroxidation and iron overload synergistically exacerbate cell damage and contribute to multi-organ failure. Sepsis-induced inflammation also boosts ROS production, further enhancing lipid peroxidation and establishing a vicious cycle that amplifies cellular damage.

### 4.3 Mitochondrial dysfunction

Mitochondria, essential for lipid and energy metabolism in eukaryotic cells, are pivotal in regulating ferroptosis (64, 65). They synthesize heme and Fe-S clusters, which are critical for ferroptosis regulation (66, 67). Iron enters mitochondria through mitoferrin 1 (SLC25A37) in erythrocytes and mitoferrin 2 (SLC25A28) in non-erythrocytes (68). Overexpression of SLC25A28 can lead to redox-active iron accumulation, increasing ferroptosis susceptibility (69). Conversely, reducing SLC25A28 activity can prevent erastin-induced ferroptosis.

Heme acts as a cofactor in metabolism and electron transfer (70). Excess heme is either exported by feline leukemia virus subtype C receptor-related protein 1B (FLVCR1B) or metabolized by heme oxygenase 1 (HO-1) into Fe^2+^, carbon monoxide (CO), and biliverdin (71). While HO-1 activation can induce ferroptosis by causing iron overload, modest upregulation of HO-1 confers protection against ferroptosis (72, 73).

Cysteine desulfurase (NFS1) is essential for Fe-S cluster synthesis, and its inhibition sensitizes cancer cells to ferroptosis (74). Increased expression of mitoNEET (CISD1) inhibits erastin-induced ferroptosis, while overproduction of NAF1 (CISD2) prevents sulfadiazine-induced ferroptosis (75, 76). The ABCB7 transporter, critical for mitochondrial iron homeostasis, also plays a role in ferroptosis regulation (77).

Overexpression of mitochondrial ferritin (FtMt) reduces erastin-induced ferroptosis by enhancing iron storage and lowering the LIP, indicating a protective role (78, 79). Voltage-dependent anion channels (VDACs), located in the outer mitochondrial membrane, facilitate chemical and energy transport and are involved in programmed cell death (80). Yagoda et al. demonstrated that knockdown of VDAC2/3 reduces erastin sensitivity in tumor cells (81). Erastin targets VDAC2/3, causing mitochondrial dysfunction and increased ROS production, suggesting that mitochondria are potential therapeutic targets for mitigating ferroptosis.

In IRI, the ischemic phase disrupts mitochondrial energy supply, decreases adenosine triphosphate (ATP) production, and causes metabolic disturbances. Although oxygen re-supply during reperfusion restores mitochondrial activity, it also dramatically increases ROS generation due to the sudden activation of the electron transport chain (82). Iron overload within mitochondria exacerbates ROS accumulation and lipid peroxidation, triggering ferroptosis. Additionally, mitochondrial membrane damage releases signaling molecules that activate downstream cell death pathways, further exacerbating tissue damage.

Sepsis, a complex systemic inflammatory response, often results in severe mitochondrial dysfunction. The release of inflammatory mediators, oxidative stress, and increased metabolic load contribute to mitochondrial damage. Disrupted iron metabolism and mitochondrial iron overload are particularly prominent in sepsis, making mitochondria a major source of ROS (83). Overproduction of mitochondrial ROS worsens lipid peroxidation, triggering ferroptosis and promoting organ dysfunction and failure. Furthermore, mitochondrial dysfunction in sepsis impairs antioxidant defenses, notably by reducing GPX4 activity. This impairs lipid peroxide reduction, fostering ferroptosis. Loss of mitochondrial function also disrupts cellular energy metabolism, diminishes the ability to manage oxidative and metabolic stress, and increases susceptibility to ferroptosis.

### 4.4 System Xc⁻-GSH-GPX4 axis

**Figure 2:**
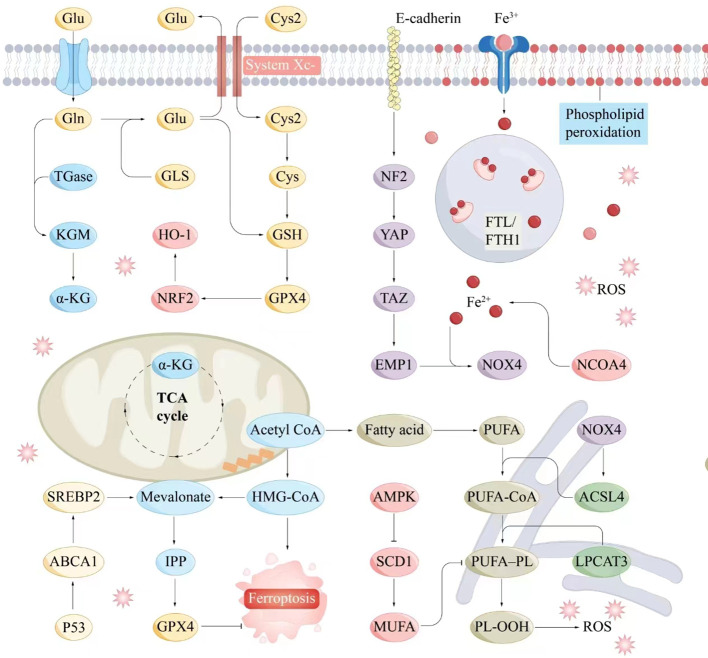
Molecular interaction diagram related to ferroptosis mechanisms. Key molecules and pathways involved in the process of ferroptosis include iron metabolism, antioxidant responses, lipid peroxidation, and related signaling pathways. Iron metabolism plays a central role in ferroptosis, as the accumulation of iron within cells can catalyze the formation of ROS through the Fenton reaction. Antioxidant Responses include GSH, GPX4, NADPH and FAD. Serve as electron donors in the regeneration of GSH and other antioxidants, supporting the cellular antioxidant response to counteract ferroptosis. Lipid peroxidation is a hallmark of ferroptosis, involving the oxidative degradation of PUFAs in cellular membranes. ABCA1, ATP-binding cassette transporter A1; Acetyl CoA, Acetyl coenzyme A; ACSL4, Acyl-CoA synthetase long-chain family member 4; AMPK, AMP-activated protein kinase; α-KG, Alpha-Ketoglutarate; Cys, Cysteine; E-cadherin, Epithelial cadherin; EMP1, Epithelial membrane protein-1; Fe^3+^, Iron ion (trivalent); Fe^2+^, Iron ion (divalent); FTL/KGM, Ferritin light chain/keratinocyte growth factor; FTH1, Ferritin heavy chain 1; Gln, Glutamine; Glu, Glutamate; GLS, Glutaminase; GPX4, Glutathione peroxidase 4; GSH, Glutathione; HMG-CoA, 3-Hydroxy-3-methylglutaryl coenzyme A; HO-1, Heme oxygenase 1; IPP, Isoprenoid pyrophosphate; LPCAT3, Lysophosphatidylcholine acyltransferase 3; Mevalonate, Mevalonic acid; MUFA, Monounsaturated fatty acid; NCOA4, Nuclear receptor coactivator 4; NF2, Neurofibromin 2; NOX4, NADPH oxidase 4; NRF2, Nuclear factor erythroid 2-related factor 2; P53, Tumor protein P53; PL-OOH, Phospholipid hydroperoxide; PUFA, Polyunsaturated fatty acid; PUFA-CoA, Polyunsaturated fatty acid coenzyme A; ROS, Reactive oxygen species; SCD1, Stearoyl-CoA desaturase-1; SREBP2, Sterol regulatory element-binding protein 2; System Xc^-^, Cystine/glutamate antiporter; TAZ, Transcription coactivator with PDZ-binding motif; TGase, Transglutaminase; TCA cycle, Tricarboxylic acid cycle; YAP, Yes1-associated transcriptional regulator.

The System Xc⁻–GSH–GPX4 axis, a crucial component of the antioxidant system, plays a pivotal role in inhibiting ferroptosis. System Xc⁻ facilitates cystine uptake, enabling GSH synthesis, which, in turn, supports GPX4 in reducing peroxides, maintaining cellular stability, and preventing ferroptosis (84). Dysregulation of this axis influences ferroptosis and contributes to various pathologies (**Figure 2**).

System Xc⁻, a sodium-independent cystine/glutamate antiporter, consists of two subunits: SLC7A11 and solute carrier family 3 member 2 (SLC3A2) (85, 86). It exports glutamate while importing cystine, initiating intracellular GSH production (85). Inhibition of SLC7A11 triggers ferroptosis, and its expression is modulated by factors such as ubiquitin aldehyde-binding protein 1 (OTUB1), BECN1, p53, and activating transcription factor 3 (ATF3) (85).

The ferroptosis inducer erastin inhibits System Xc⁻, depleting GSH and promoting lipid peroxidation (11). Mutant p53 suppresses SLC7A11 expression, increasing lipid peroxide accumulation and inducing ferroptosis, although p53 can also upregulate GSH levels through p21 activation (87, 88). ATF3 and ATF4 regulate System Xc⁻ activity, either promoting or mitigating ferroptosis depending on context (89, 90). Nrf2 enhances SLC7A11 transcription, boosting GSH synthesis, while its loss amplifies ferroptosis susceptibility (91).

Phosphorylated BECN1 inhibits SLC7A11, inducing ferroptosis (92). In liver cancer, the ferroptosis-inducing drug sorafenib is effective at low doses but often encounters resistance (93, 94). DJ-1 suppression can enhance sorafenib efficacy (94). Additionally, dipeptidase-1 (DPEP1) exacerbates oxidative stress, increasing ferroptosis susceptibility (95).

GPX4, essential for lipid peroxide detoxification, relies on selenium and GSH. Selenium not only supports GPX4 expression but also plays a role in immune responses; its deficiency results in embryonic lethality (96, 97). The mevalonate pathway further regulates GPX4 expression and ferroptosis sensitivity (98, 99). Ferroptosis inducers such as Ferroptosis-Inducer-56 (FIN56) and RSL3 target GPX4 (87, 100). Additionally, ferroptosis suppressor protein-1 (FSP1), identified through CRISPR screening, inhibits ferroptosis independently of GPX4, highlighting the importance of the Coenzyme Q10 (CoQ10)–FSP1–NAD(P)H axis (101, 102).

In IRI, dysfunction of the System Xc⁻–GSH–GPX4 axis exacerbates oxidative stress and cellular damage. Reperfusion generates large amounts of ROS, while impaired System Xc⁻ function limits GSH replenishment, rendering GPX4 inactive. The resulting lipid peroxidation intensifies cell injury and ferroptosis. Similarly, in sepsis, extensive inflammation, oxidative stress, and immune activation disrupt this axis. System Xc⁻ inhibition reduces GSH availability, weakening cellular defenses against oxidative damage. Additionally, reduced GPX4 activity prevents lipid peroxidation resolution, leading to membrane instability and ferroptosis, which accelerates the progression of multiple organ failure.

### 4.5 CoQ10-FSP1-NADH axis

CoQ10, also known as ubiquinone, is a lipophilic metabolite synthesized through the mevalonate pathway. Comprising an isoprenoid polymer and a benzoquinone ring, CoQ10 is indispensable for mitochondrial energy production (103). In its reduced form, it functions as a potent antioxidant, neutralizing free radicals generated by lipid peroxidation and thereby preventing ferroptosis (104).

CoQ10 is synthesized from acetyl-coenzyme A through the MVA pathway and is regulated at both transcriptional and translational levels (105). FSP1, initially identified as p53-responsive gene 3 (PRG3) and associated with p53-mediated apoptosis, shares sequence similarities with apoptosis-inducing factor (AIF) (106, 107).

FSP1, a flavoprotein oxidoreductase, interacts with DNA and uses NADPH to reduce ubiquinone to ubiquinol outside mitochondria, thereby inhibiting lipid peroxidation independently of GPX4 and GSH (101, 102). Elevated FSP1 expression enhances resistance to ferroptosis, a process regulated by murine double minute 2 (MDM2) and murine double minute X (MDMX) through peroxisome proliferator-activated receptor α (PPARα) (108). Plasma-activating mediators (PAM) reduce FSP1 expression, triggering ferroptosis in lung cancer, while the compound NPD4928 inhibits FSP1 and promotes ferroptosis (106, 107). Additionally, FSP1 reduces vitamin K to vitamin K hydroquinone (VKH2), which scavenges free radicals and prevents ferroptosis (109). The CoQ10-FSP1-NADH axis underscores FSP1 as a distinct ferroptosis inhibitor, separate from GPX4.

In IRI, the ischemic phase is marked by inadequate oxygen supply, reduced cellular metabolism, and diminished ATP and NADH production. Upon reperfusion, oxygen is reintroduced, rapidly generating ROS and inducing severe oxidative stress. FSP1 mitigates the expansion of lipid free radicals by reducing CoQ10 to panthenol using NADH, thus decreasing oxidative stress, limiting cell damage, and preventing ferroptosis. In sepsis, persistent inflammation and immune activation lead to excessive ROS production and enhanced lipid peroxidation. FSP1 function or expression may be impaired, exacerbating cellular antioxidant demands. The CoQ10-FSP1-NADH axis provides a crucial antioxidant pathway, removing lipid peroxides, maintaining membrane integrity, and reducing ferroptosis, thereby preventing multi-organ dysfunction to some degree.

### 4.6 DHODH-CoQH2 axis

The inner mitochondrial membrane hosts dihydroorotate dehydrogenase (DHODH), a key enzyme in pyrimidine ribonucleotide synthesis. Recent studies suggest that DHODH also functions as an antioxidant, similar to FSP1, by reducing CoQ10 and modulating ferroptosis independently of mitochondrial GPX4, highlighting its role in oxidative stress management and cell survival (110). DHODH catalyzes the oxidation of dihydroorotate (DHO) to orotate (OA) and facilitates the conversion of ubiquinone (CoQ) to ubiquinol (CoQH2), integral to respiratory complexes and electron transfer in oxidative phosphorylation (111). High DHODH expression confers ferroptosis resistance, while low expression increases susceptibility. DHODH functions independently of FSP1, working alongside GPX4 to prevent mitochondria-associated ferroptosis (110). Wang et al. demonstrated that DHODH provides an independent antioxidant pathway, complementing GPX4 in ferroptosis regulation (112). DHODH inhibitors, such as SA771726 (a leflunomide metabolite) and Brequinar sodium (BQR), are important in cancer therapy (113).

In IRI, the initial ischemic phase disrupts mitochondrial function and blocks the electron transport chain. The subsequent restoration of oxygen during reperfusion triggers the rapid generation of ROS. The DHODH-CoQH2 axis mitigates this damage by maintaining mitochondrial redox balance, reducing ROS-induced cellular damage, preventing lipid peroxidation, and inhibiting ferroptosis. In sepsis, sustained oxidative stress from systemic inflammation results in mitochondrial dysfunction and enhanced lipid peroxidation. The DHODH-CoQH2 axis alleviates oxidative stress, supports mitochondrial and cellular membrane integrity through continuous CoQH2 supply, and inhibits ferroptosis, thereby protecting cells and reducing organ dysfunction.

### 4.7 GCH1-BH4 axis

Tetrahydrobiopterin (BH4), synthesized by GTP cyclohydrolase 1 (GCH1) from guanosine triphosphate, plays a crucial role in limiting intracellular CoQ and ROS accumulation, preventing ferroptosis. It specifically inhibits the consumption of phospholipids containing polyunsaturated fatty acyl tails (114). While BH4 is essential for various enzymes, its role in ferroptosis inhibition is newly discovered. As an antioxidant, BH4 reduces lipid peroxidation, traps free radicals, and safeguards cells, particularly under conditions where GPX4 is inhibited. BH4’s protective effects are enhanced through regeneration by dihydrofolate reductase (DHFR) (115).

Kraft et al. demonstrated that GCH1 increases BH4 synthesis, facilitates lipid remodeling, and reduces lipid peroxidation, thereby inhibiting ferroptosis (114). Additionally, BH4 aids in converting phenylalanine to tyrosine, which is subsequently transformed into 4-OH-benzoate, a precursor for CoQ10 synthesis, further promoting ferroptosis inhibition. Studies underscore the GCH1-BH4 axis as critical in regulating tumor cell susceptibility to ferroptosis by modulating iron metabolism and mitigating ferritin toxicity through inhibition of NCOA4-mediated ferritin autophagy in colorectal cancer (115, 116). Moreover, Nrf2 has been shown to activate GCH1, promoting BH4 synthesis and protecting cells from oxidative stress, particularly in radiation-induced damage (117). The GCH1-BH4 axis thus represents a major regulatory pathway for ferroptosis, offering a strategy independent of the GSH/GPX4 system.

In IRI, the GCH1-BH4 axis enhances antioxidant defense by boosting BH4 synthesis, reducing ROS damage to cellular membranes, and preventing ferroptosis-induced cell death. In sepsis, this axis helps minimize cellular damage, stabilizes the intracellular environment, and reduces lipid peroxidation, thereby lowering the risk of organ dysfunction.

### 4.8 p53

The tumor suppressor gene p53 plays a central role in managing cellular stress, including ribosomal stress, hypoxia, and DNA damage. Mutations in p53 and associated signaling pathways are key drivers of tumorigenesis (118). Ferroptosis, a process regulated by p53, is modulated through both transcription-dependent and independent mechanisms (119). p53 promotes ferroptosis by downregulating SLC7A11 expression through Ubiquitin-specific proteinase 7 (USP7), reducing histone H2B monoubiquitination (2). It binds to the SLC7A11 promoter, thereby affecting GPX4 activity and inducing lipid peroxidation (60). However, p53 does not significantly impact GSH levels or GPX4 function, likely due to the activation of Glutaminase 2 (GLS2), TIGAR (p53-induced glycolysis-regulated phosphatase), and CDKN1A (60). p53 activation increases expression of TfR1 and SLC25A28, leading to iron accumulation and heightened sensitivity to ferroptosis (69, 120).

Additionally, p53 targets Spermidine/spermine N1-acetyltransferase 1 (SAT1), enhancing ALOX15 activity and promoting lipid peroxidation under oxidative stress (121). In contrast, p53 can inhibit ferroptosis by enhancing GSH levels through the p53-p21 pathway and regulating ROS through Parkin (122). The p53-DPP4 interaction within the nucleus suppresses Dipeptidyl peptidase-4 (DPP4) and inhibits NADPH oxidase (NOX1), offering protection against ferroptosis (108). DPP4 inhibitors, such as vildagliptin, may reduce the anticancer efficacy of ferroptosis activators (88). MDM2 ubiquitinates p53, but MDM2 inhibitors like nutlin-3 can delay ferroptosis (122). p53 also inhibits erastin-induced ferroptosis by inducing CDKN1A in specific cell types (122). Overall, p53’s regulation of ferroptosis is complex, influencing lipid, energy, and iron metabolism in a cell-type and context-dependent manner.

In IRI, p53 enhances cellular susceptibility to ferroptosis by inhibiting antioxidant defenses and promoting lipid peroxidation. This process may be protective by clearing damaged cells in ischemia-reperfusion, but it can also exacerbate tissue damage and dysfunction. In sepsis, p53 similarly mediates oxidative stress-induced ferroptosis. Inflammation-driven persistent oxidative stress activates p53, which suppresses antioxidant systems and drives lipid peroxidation, thus triggering ferroptosis. While this cell death aids in clearing damaged cells and pathogens, excessive ferroptosis can damage tissues and organs, exacerbating sepsis-related organ failure.

### 4.9 Nrf2

Cloned in 1994, Nrf2 is a member of the basic leucine zipper (bZIP) transcription factor family, including cap “n” collar (CNC) proteins. It is crucial for maintaining redox homeostasis and preventing oxidative stress by regulating genes associated with oxidative stress (123). During ferroptosis, Nrf2 activates cytoprotective genes involved in iron metabolism and redox signaling.

Nrf2 promotes the transcription of antioxidant-related genes by binding to antioxidant response elements (AREs) (124). Upon oxidative stress, Nrf2 dissociates from Kelch-like ECH-associated protein 1 (KEAP1) and translocates to the nucleus, triggering the expression of genes linked to downstream AREs. These include HO-1, quinone oxidoreductase 1 (NQO1), Fpn1, ferritin heavy chain 1 (FTH1), ferritin light chain (FTL), and Fpn1, which collectively promote GSH synthesis and limit ROS production (125, 126).

The Nrf2/KEAP1 pathway enhances the expression of System Xc⁻, which targets SLC7A11, indicating that SLC7A11 may mitigate Nrf2-mediated ferroptosis (127). Nrf2 has been shown to inhibit ferroptosis in mice with folate-induced kidney injury through the regulation of GPX4. It also facilitates NADPH production in the pentose phosphate pathway (99). Metallothionein 1G (MT1G), an Nrf2 target gene, helps hepatocellular carcinoma (HCC) cells resist ferroptosis (128). Additionally, Nrf2-driven ATP-binding cassette subfamily C member 1 (ABCC1/MRP1) promotes GSH efflux, potentially influencing ferroptosis regulation (129).

Nrf2 serves as a critical negative regulator of ferroptosis within complex molecular signaling networks. However, further preclinical and clinical research is essential to fully elucidate Nrf2’s role in ferroptosis resistance and the therapeutic potential of nuclear factor erythroid 2-like 2 (NFE2L2) inhibitors.

In IRI, Nrf2 mitigates ferroptosis-induced cell damage by upregulating antioxidant enzymes and related protective proteins, thereby reducing ROS accumulation and lipid peroxidation. This protective effect is crucial for minimizing tissue damage and facilitating repair after reperfusion. In sepsis, Nrf2 effectively reduces excessive ROS generation and lipid peroxidation, regulating antioxidant gene expression to preserve cell survival and function. Nrf2 activation stabilizes the intracellular environment, preventing multiple organ injuries induced by ferroptosis and improving sepsis prognosis.

### 4.10 YAP1/WWTR1

The Hippo signaling pathway depends on two key effector proteins: WW domain-containing transcriptional regulator 1 (WWTR1/TAZ) and Yes1-associated transcriptional regulator (YAP1, also known as YAP), which control organ size, tissue homeostasis, and cancer growth (130, 131). Targeting YAP1/WWTR1 has therapeutic potential in cancer treatment and regenerative medicine (130, 131). These proteins act as biomechanical and morphological sensors, responsive to the cellular environment.

Cadherin 1 (CDH1/E-cadherin)-dependent cell adhesion enhances ferroptosis resistance through the Hippo pathway (55). E-cadherin activates the tumor suppressor neurofibromin 2 (NF2) in epithelial cells, reducing YAP1 activity and ferroptosis (55). Conversely, inhibiting Hippo pathway effectors such as NF2/merlin, large tumor suppressor kinases 1 (LATS1), and 2 (LATS2) reinstates cancer cell susceptibility to ferroptosis, particularly at high cell densities (55).

WWTR1 modulates angiopoietin-like 4 (ANGPTL4) and epithelial membrane protein 1 (EMP1) levels, while YAP1 influences transferrin receptor complexes (TFRC) and ACSL4 translation, promoting lipid peroxidation and iron accumulation, thereby inducing ferroptosis (55, 132). In conclusion, YAP1 and WWTR1 are key transcriptional regulators of ferroptosis, though further *in vivo* studies are necessary.

During IRI, YAP1/WWTR1 activation helps cells manage oxidative stress and attenuate ferroptosis by promoting antioxidant gene expression and defense mechanisms. Modulating YAP1/WWTR1 activity may alleviate tissue injury following ischemia-reperfusion and protect cells from lipid peroxidation and ferroptosis. In sepsis, YAP1/WWTR1 acts as a regulator, helping cells cope with excessive ROS generation and oxidative damage (133). Its activation promotes antioxidant gene expression, metabolic regulation, and reduces ferroptosis-associated apoptosis and histological damage, thereby preserving organ function and minimizing multiple organ damage caused by sepsis.

### 4.11 Activating transcription factor

ATF, part of the cAMP response element-binding protein 2 family, which includes ATF3 and ATF4, is critical for pathways involving autophagy, oxidative stress, and inflammation (134). Endoplasmic reticulum (ER) stress stimulates ATF expression, influencing metabolism and oxidative balance (135).

In human fibrosarcoma cells, ATF3 directly represses SLC7A11 during erastin-induced ferroptosis (89). Conversely, ATF4 activation enhances SLC7A11 expression, modulating ferroptosis (90). Overexpression of DNA damage-inducible transcript 3 (DDIT3/CHOP) in human glioblastoma, pancreatic, and Burkitt’s lymphoma cells links ATF4 to ferroptosis-associated diseases, such as Burkitt’s lymphoma and diabetic myocardial IRI (136, 137).

ATF4’s target gene, ChaC glutathione-specific γ-glutamyl cyclotransferase 1 (CHAC1), catalyzes GSH degradation, increasing the susceptibility of triple-negative breast cancer cells to ferroptosis (138). In contrast, heat shock 70 kDa protein 5 (HSPA5), an ER chaperone, mitigates ferroptosis by stabilizing GPX4 (139). These findings underscore ATF4’s involvement in ferroptotic cell death through complex signaling pathways, highlighting the need for further research on its precise role in ferroptosis (90).

In IRI, ATF4 is activated under ER stress and regulates cellular responses to protein misfolding, crucial for maintaining cellular function and preventing ferroptosis. Enhancing ATF4 activity has been shown to improve cellular tolerance to oxidative stress induced by ischemia-reperfusion (I/R), reducing cell death associated with ferroptosis (140). ATF4 also protects against oxidative damage and modulates immune responses in sepsis by regulating cellular redox balance. Its activation increases the expression of antioxidant proteins, alleviates lipid peroxidation, and prevents ferroptosis caused by excessive ROS accumulation, thereby safeguarding organs and tissues.

### 4.12 Hypoxia-inducible factor

HIF plays a pivotal role in cellular responses to fluctuating oxygen levels. It is composed of a stable β component, aryl hydrocarbon receptor nuclear translocator (ARNT1/HIF1B), and a labile α subunit, endothelial PAS domain protein 1 (EPAS1/HIF2A), which together drive the expression of hypoxia-inducible factor 1α (HIF1A) and HIF3α, forming a heterodimeric transcription factor (141).

Under normoxic conditions, HIF1α and EPAS1 are hydroxylated by EGLN enzymes, which target them for degradation via the Von Hippel-Lindau (VHL) E3 ubiquitin ligase complex (142). HIF modulates ferroptosis in tumor cells through two mechanisms: EGLN serves as a key target for iron chelators like deferoxamine, which stabilize HIF by inhibiting EGLN (143).

Hypoxia induces HIF1α expression in HT-1080 fibrosarcoma cells, leading to increased transcription of fatty acid binding proteins 3 (FABP3) and 7 (FABP7), enhancing lipid uptake and storage, which inhibits ferroptosis (144). In contrast, EPAS1 upregulates hypoxia-induced lipid droplet-associated proteins (HILPDA/HIG2) in clear-cell carcinoma cells, promoting ferroptosis through lipid peroxidation and increased PUFAs synthesis (145).

These findings suggest that tumor type influences HIF’s regulation of ferroptosis. Understanding HIF’s role across different cancers could inform innovative therapeutic strategies for cancer treatment and prevention.

HIF is activated during ischemia in IRI, promoting cellular adaptation to hypoxic conditions and mitigating ROS damage by regulating antioxidant genes and metabolic pathways during reperfusion. This regulation inhibits ferroptosis. By controlling glycolysis and other metabolic pathways, HIF enhances cellular energy production and antioxidant capacity, which is critical for responding to hypoxia and oxidative stress. In sepsis, HIF modulates metabolic and inflammatory responses by regulating hepcidin and ferroportin expression, thereby maintaining intracellular iron homeostasis. This reduces free iron accumulation, limits excessive ROS generation through the Fenton reaction, and promotes antioxidant enzyme expression, aiding in ROS scavenging and preventing oxidative stress-induced ferroptosis.

## 5 Role of ferroptosis in critical illness

### 5.1 IRI

**Figure 3:**
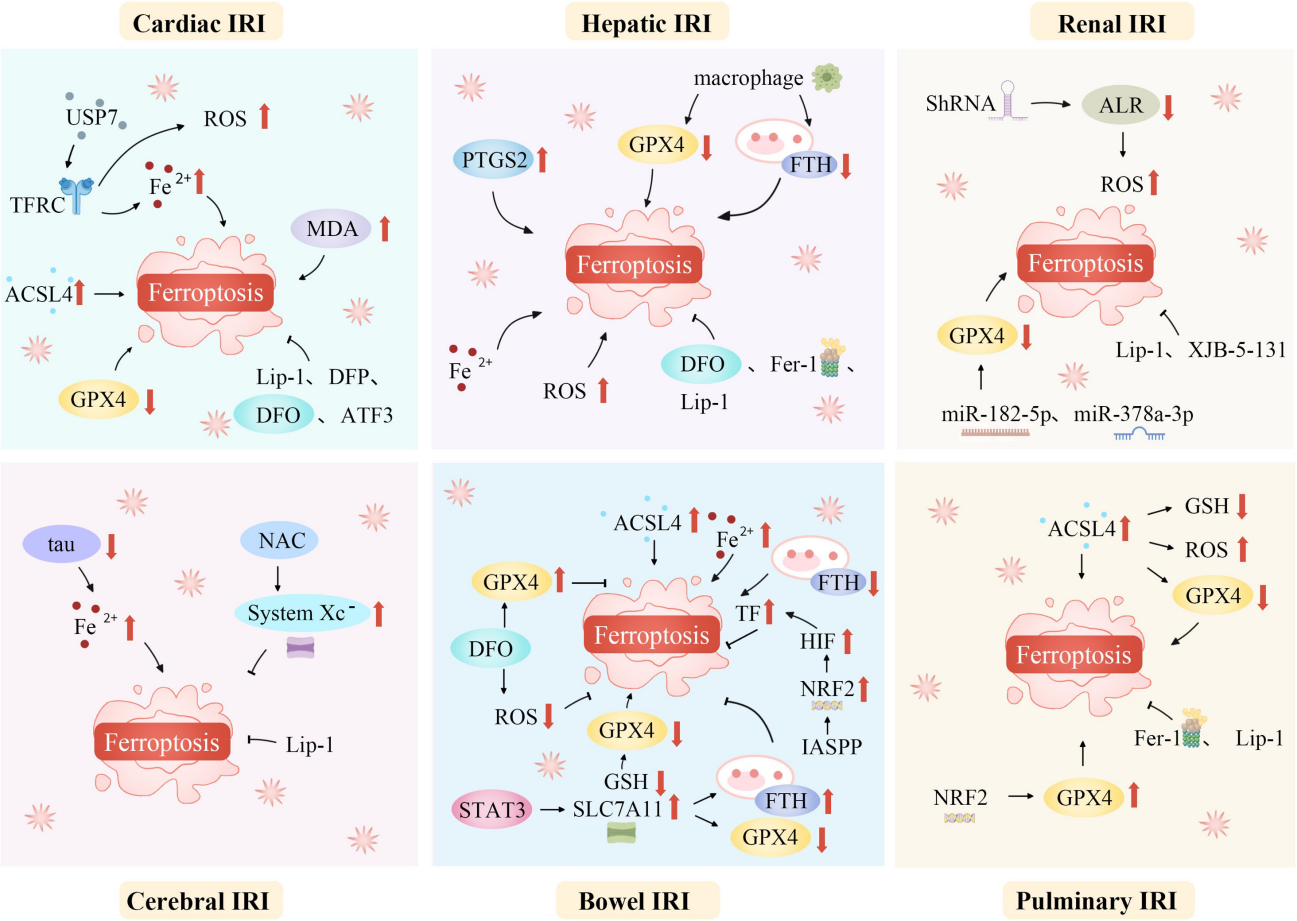
Ferroptosis mediates a variety of ischemia-reperfusion injuries. Ferroptosis, a unique regulated cell death form, mediates various IRIs. During ischemia, restricted blood supply causes hypoxia, disrupting metabolism and leading to intracellular changes like ROS generation in mitochondria’s electron transport chain. Reperfusion restores blood flow but worsens oxidative stress. Increased ROS overwhelms antioxidant systems like GPX4, triggering ferroptosis. Abundant tissue iron ions in the ROS-influenced Fenton reaction generate hydroxyl radicals, initiating lipid peroxidation. This is key in ferroptosis. In organs like the heart, brain, kidneys, and liver, ferroptosis-caused lipid metabolism disruption and peroxide accumulation damage cell membranes, causing cell death. Thus, ferroptosis-mediated damage in ischemia-reperfusion contributes to tissue dysfunction and organ failure. ACSL4, Acyl-CoA synthetase long-chain family member 4; ALR, Augmenter of liver regeneration; ATF3, Activating transcription factor 3; DFO, Deferoxamine; DFP, Deferiprone; Fe^2+^, Iron ion; Fer-1, Ferrostatin-1; FTH, Ferritin heavy chain; GPX4, Glutathione peroxidase 4; GSH, Glutathione; HIF, Hypoxia-inducible factor; IASPP, Inhibitor of apoptosis stimulating protein of p53; Lip-1, Liproxstatin-1; IRI, Ischemia-reperfusion injury; NAC, N-acetylcysteine; NRF2, Nuclear factor erythroid 2-related factor 2; PTGS2, Prostaglandin-endoperoxide synthase 2; ROS, Reactive oxygen species; ShRNA, short hairpin RNA lentivirals; SLC7A11, Solute carrier family 7 member 11; STAT3, Signal transducer and activator of transcription 3; System Xc^-^, Cystine/glutamate antiporter; TF, Transferrin; TFRC, Transferrin receptor complexes; USP7, Ubiquitin-specific proteinase 7.

IRI is a common, life-threatening clinical complication with significant local and systemic effects. Recent studies suggest that ferroptosis plays a role in the pathophysiology of IRI across various organs, including the brain, intestines, liver, heart, and kidneys (20, 72, 146, 147). Modulating lipid peroxidation and iron levels influences susceptibility to ferroptosis, thereby affecting IRI outcomes (**Figure 3**).

#### 5.1.1 Cardiac IRI

Cardiac IRI and myocardial infarction are prevalent cardiovascular conditions associated with poor prognosis and limited early intervention options. Before the identification of ferroptosis, studies indicated that mitochondrial overexpression of GPX4 had cardioprotective effects against IRI (148). Ferroptosis predominantly occurs during the cardiac reperfusion phase, rather than during ischemia. Prolonged reperfusion results in a decline in GPX4 levels, while ferroptosis markers, including ACSL4, iron, and MDA, increase (149).

In 2015, Gao et al. first highlighted the role of ferroptosis in cardiac I/R (150). Iron concentrations increase near coronary blood flow after prolonged myocardial ischemia (151). Following cardiac IRI, cardiomyocytes become more vulnerable to ferroptosis as excess iron is absorbed by lipid peroxides through the Fenton reaction and enters the cells. Moreover, cardiomyocytes exposed to high iron levels become more susceptible to oxidative stress due to increased ROS, which, in turn, triggers ferroptosis.

Iron overload significantly contributes to myocardial cell injury, with lipid peroxidation playing a pivotal role in oxidative IRI (152, 153). In response to I/R, the heart exhibits increased production of ferritin heavy chain (FTH) and FTL, indicating elevated non-heme iron levels in ischemic myocardial tissue (72). Cardiomyocytes treated with erastin or RSL3 show decreased Fe^2+^, ROS, MDA, and cell death levels when ATF3 is overexpressed, preventing ferroptosis in these cells (152).

An *in vitro* study revealed that phospholipid oxidation products negatively affect cardiomyocyte viability during I/R (154). Oxidized lipidomic analysis identified oxidized phosphatidylethanolamine as a specific indicator of ferroptosis, providing direct evidence for cardiac ferroptosis (147). Moreover, I/R-treated hearts demonstrated the formation of oxidized phosphatidylethanolamine in their mitochondria, underscoring the role of cardiac mitochondria in lipid peroxide generation and ferroptosis signaling (155). Additionally, inhibiting or knocking down USP7 suppresses TFRC expression, reducing myocardial IRI primarily by decreasing iron levels, lipid peroxidation, and ferroptosis (120).

Li et al. investigated the *in vivo* inflammatory responses triggered by IRI following heart transplantation. Their results demonstrated that ferroptosis facilitated endothelial cell adhesion in cardiac tissues via the type I interferon/TLR4/Trif signaling pathway, which in turn promoted neutrophil recruitment to the injured myocardium (147). Moreover, using a coronary artery ligation model to induce myocardial IRI, they found that suppression of ferroptosis improved heart function and reduced infarct size.

#### 5.1.2 Hepatic IRI

Hepatic IRI poses a significant challenge in liver transplantation, as it induces an immune response and inflammatory cascade that impairs donor liver function and worsens recipient prognosis (154). In pediatric living donor liver transplants (LTx), donor iron burden has been identified as an independent risk factor for liver IRI (156). Iron overload, lipid peroxidation, and the upregulation of Prostaglandin-endoperoxide synthase 2 (PTGS2)—a marker of ferroptosis—are key features of IRI progression in the liver.

*In vitro* studies have shown that hepatic macrophages play a role in ferroptosis during IRI, with macrophage co-culture leading to ferroptosis through downregulation of GPX4 and FTH1 (157). Additionally, mice subjected to a high-iron diet exhibited aggravated IRI, which was mitigated by treatment with DFO104.

#### 5.1.3 Renal IRI

Ferroptosis is firmly linked to renal IRI, where it significantly contributes to the pathophysiological processes associated with the condition. The tubular manifestation of ferroptosis plays a crucial role in the progression of renal IRI (158).

In the context of renal injury and repair, augmenter of liver regeneration (ALR) has been shown to influence ferroptosis (159). Blocking ALR with short hairpin RNA (shRNA) exacerbates the condition, increasing ROS production, mitochondrial damage, and ferroptosis (159). Using a rat model of renal IRI, Ding et al. demonstrated that miR-182-5p and miR-378a-3p directly bind to the 3′-untranslated region of GPX4 mRNA, suppressing GPX4 expression and promoting ferroptosis (160).

Reducing iron-related antioxidants and administering iron chelators can improve renal function in patients with renal IRI (161). These findings underscore the close association between ferroptosis and renal IRI, involving multiple regulatory mechanisms.

#### 5.1.4 Cerebral IRI

The pathophysiological mechanisms underlying cerebral IRI are multifactorial, involving factors such as heightened inflammatory responses, calcium overload, oxidative damage, excessive release of excitatory amino acids, and processes leading to apoptosis or necrosis (162). Most research on ferroptosis in cerebral IRI focuses on oxygen-glucose deprivation/reoxygenation models and animal models of focal or global brain injury.

In focal cerebral IRI, a middle cerebral artery occlusion (MCAO) model, based on the traditional MCAO technique, revealed reduced ferritin expression. In this model, ferritin overexpression modulated p53/SLC7A11-mediated ferroptosis, improving memory and cognitive function (163). Significantly suppressed tau expression, characteristic of cerebral I/R, is linked to Alzheimer’s disease and promotes iron efflux. This impairs iron transport to the extracellular space, increases intracellular iron levels, and induces neurotoxicity and ferroptosis, thereby exacerbating neuronal injury (164, 165).

Due to the complex nature of cerebral IRI and the incomplete understanding of ferroptosis mechanisms, it is critical to consider the interplay between multiple factors when studying the effects of ferroptosis on cerebral IRI. Results from single-perspective studies often fail to yield conclusive outcomes in broader animal models or clinical settings.

#### 5.1.5 Bowel IRI

The intestine, particularly the small intestine, is one of the most vulnerable organs to IRI, which typically results from a sudden decrease in blood flow followed by reoxygenation upon restoration of circulation (165). This condition is associated with various forms of regulated cell death (RCD), including ferroptosis, apoptosis, necroptosis, and autophagy (166, 167). Lipid peroxidation and ROS generation play a pivotal role in initiating and completing ferroptosis in intestinal IRI (168).

A crucial enzyme, ACSL4, regulates lipid content and is vital for initiating ferroptosis (57). Li et al. demonstrated that intestinal IRI leads to decreased levels of GPX4, ferritin heavy chain, and GSH, while increasing ACSL4 and iron levels. This process results in ferroptosis of intestinal histiocytes (146). Inhibiting ACSL4, a key ferroptosis controller, through medicinal and biological approaches successfully prevented lipid peroxidation and ferroptosis. This intervention also alleviated cell injury and dysfunction of the intestinal barrier caused by IRI (57, 146).

Ferroptosis in intestinal IRI has been further investigated through modulation of SLC7A11, which influences intestinal IRI-induced acute lung injury (ALI) (169). The inhibitor of apoptosis-stimulating protein of p53 (IASPP) mitigates ferroptosis in intestinal IRI by activating the Nrf2-HIF1-TF signaling pathway, thereby reducing ALI (170).

Additionally, intestinal microbiota and its metabolites can inhibit ferroptosis in intestinal IRI. A potential mechanism involves capsaicin ester, a metabolite of the microbiota, which activates transient receptor potential cation channel subfamily V member 1 (TRPV1) to enhance GPX4 expression, thus suppressing ferroptosis (169).

#### 5.1.6 Pulmonary IRI

Pulmonary IRI remains a complex and not fully understood condition. Despite significant recent research, our understanding of the mechanisms underlying pulmonary IRI and ferroptosis is still in its early stages, warranting further comprehensive investigation.

Inhibition or knockdown of ACSL4 prevents I/R-induced lung injury by reducing lipid peroxidation and elevating GSH and GPX4 levels, thereby mitigating ferroptosis (171). These findings suggest that increased Nrf2 expression protects against lipid peroxidation-induced injury in I/R by upregulating GPX4 and SLC7A11 (172). Future research should focus on elucidating the mechanisms of pulmonary IRI to identify more precise therapeutic targets and develop effective clinical treatments.

### 5.2 Sepsis

**Figure 4:**
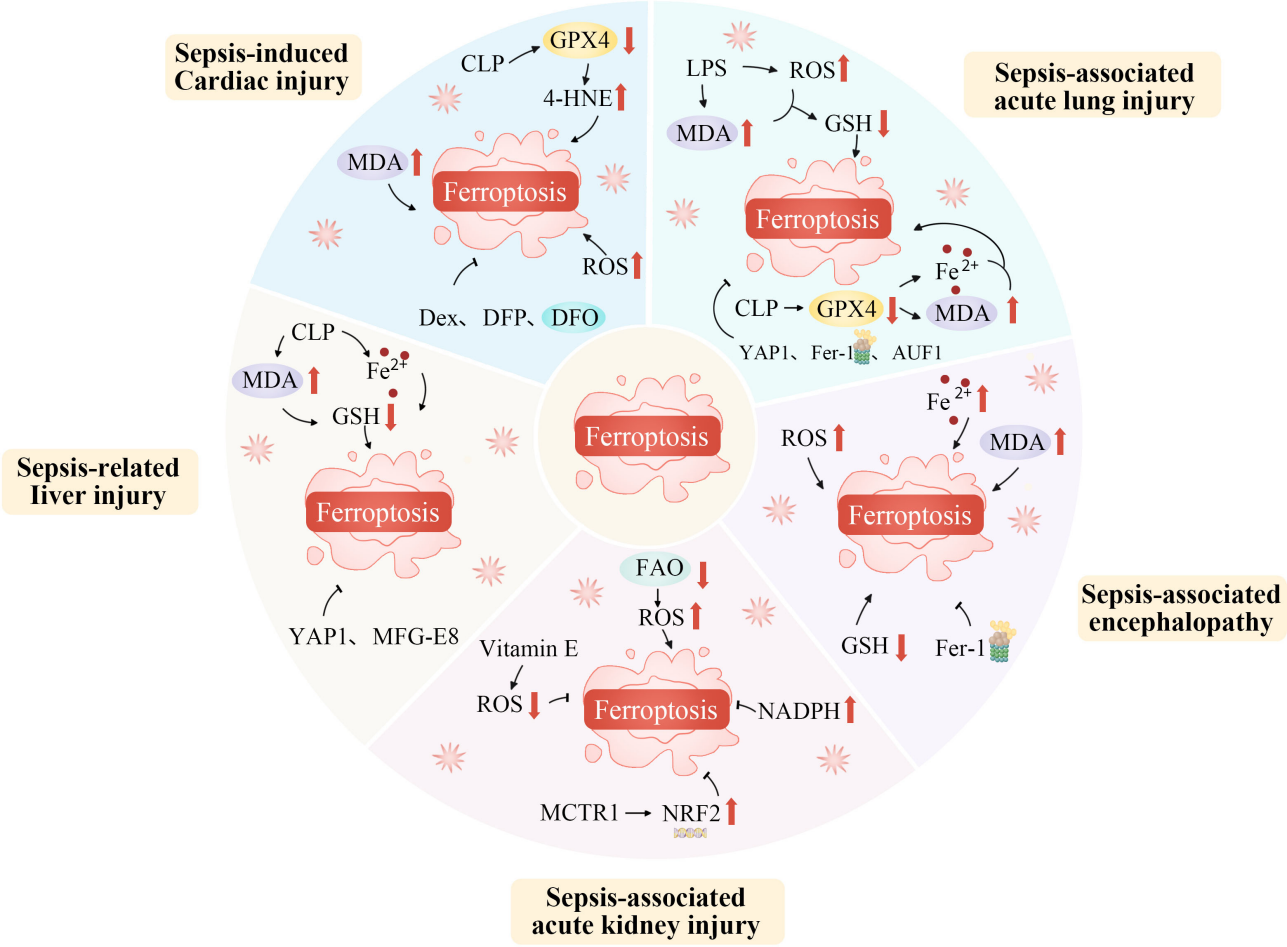
Ferroptosis plays an important role in the occurrence and progression of sepsis. Ferroptosis, as a specific form of regulated cell death, plays an indispensably important role in the occurrence and progression of sepsis. During the development of sepsis, the complex pathophysiological environment, including excessive inflammation, oxidative stress, and disrupted iron metabolism, creates favorable conditions for ferroptosis. The dysregulation of iron homeostasis within cells leads to an accumulation of iron ions, which act as catalysts for lipid peroxidation. Inflammatory cytokines and reactive oxygen species generated during sepsis can further disrupt the antioxidant defense system within cells, thereby exacerbating lipid peroxidation. This process of ferroptosis in turn aggravates tissue damage, impairs organ function, and promotes the continuous progression of sepsis, ultimately having a profound impact on the prognosis of patients with this severe condition. AUF1, AU-rich element RNA binding factor 1; CLP, Cecal ligation and puncture; Dex, Dexmedetomidine; DFO, Deferoxamine; DFP, Deferiprone; FAO, Fatty acid oxidation; Fe^2+^, Iron ion; Fer-1, Ferrostatin-1; GSH, Glutathione; GPX4, Glutathione peroxidase 4; LPS, Lipopolysaccharide; MCTR1, Maresin conjugates in tissue regeneration-1; MDA, Malondialdehyde; MFG-E8, Epidermal growth factor 8; NADPH, Nicotinamide adenine dinucleotide phosphate; NRF2, Nuclear factor erythroid 2-related factor 2; ROS, Reactive oxygen species; YAP1, Yes1 associated transcriptional regulator; 4-HNE, 4-hydroxynonenal.

Sepsis is a syndrome in which the host’s response to infection becomes dysregulated, potentially leading to multiple organ failure and life-threatening dysfunction (173). It involves complex processes such as activation of the innate immune system, inflammatory cascades, procoagulant and antifibrinolytic pathways, altered cellular metabolism, and modifications to signaling pathways. However, the central pathophysiological change in sepsis is acquired immunological dysfunction (174, 175). Ferroptosis, strongly linked to immune cell dysfunction, oxidative stress, and metabolic disturbances, significantly impacts the immune response, influencing the onset and progression of sepsis (**Figure 4**) (176).

#### 5.2.1 Sepsis-induced cardiac injury

Sepsis-induced heart failure, known as septic cardiomyopathy, significantly raises mortality rates (177). Previous reviews have highlighted ferroptosis as a key component of the pathogenesis of septic cardiomyopathy (178). Lin et al. identified lipid peroxidation products, including malondialdehyde, 4-hydroxynonenoic acid, and lipid ROS, in H9c2 myofibroblasts and a mouse model of lipopolysaccharide (LPS)-induced septic cardiomyopathy. The authors observed morphological signs of LPS-induced mitochondrial damage, consistent with microstructural alterations characteristic of ferroptosis in mitochondria (179).

In a mouse model of CLP-induced septic heart injury, Wang et al. found that CLP elevated the cardiac injury marker troponin I (TNI). Moreover, CLP significantly reduced levels of GSH and GPX4, while increasing concentrations of HO-1, TfR1, caspase-3, inducible nitric oxide synthase, and Fe^2+^ (180).

Ferroptosis was also found to heighten atrial vulnerability in the LPS-induced sepsis model, which contributed to the onset of new atrial fibrillation. Conversely, knocking down Fpn or administering ferroptosis inhibitors mitigated atrial fibrillation development (181). These findings suggest that ferroptosis plays a crucial role in sepsis-related heart damage and that targeting ferroptosis may offer therapeutic potential.

#### 5.2.2 Sepsis-associated lung injury/ARDS

Acute respiratory distress syndrome (ARDS), a common consequence of sepsis, is also linked to ferroptosis (182, 183). Xu et al. emphasized the role of ferroptosis in the pathophysiology of SALI (184). In a study of LPS-induced septic lung injury in A549 cells, LPS was shown to elevate ROS, iron, and MDA levels, while reducing GSH in alveolar cells (185). Additionally, in a murine model of ALI caused by sepsis via cecal ligation, ferroptosis marker GPX4 expression was downregulated, while MDA and iron ion levels increased.

YAP1 mitigates SALI by inhibiting ferroptosis via ferritin phagocytosis. In a CLP-induced murine model, YAP1 knockout exacerbated SALI, as indicated by increased expression of mitochondrial membrane proteins SFXN1 and NCOA4, alongside decreased levels of GPX4, FTH1, and SLC7A11 (186).

The KEAP1-Nrf2/HO-1 pathway plays a protective role in the pathogenesis of SALI. Activation of Nrf2 by American ginseng phenol has been shown to ameliorate septic ALI by upregulating HO-1 expression (187). Furthermore, mucin and itraconazole protect against macrophage ferroptosis and alleviate SALI by enhancing GPX4 expression via Nrf2-mediated pathways (188, 189). Overall, ferroptosis is a critical factor in SALI development, representing a promising target for both preventive and therapeutic interventions.

#### 5.2.3 Sepsis-related liver injury

Emerging evidence highlights ferroptosis as a key mechanism in hepatocellular injury during sepsis. In a CLP-induced sepsis model, mice exhibited hepatic ferroptosis, characterized by elevated iron levels, increased MDA, and reduced GSH.

YAP1 prevents ferroptosis driven by ferritin phagocytosis, offering protection against septic liver injury, while its deficiency worsens liver damage during sepsis (190). Irisin has also been shown to reduce ferroptosis in LPS-treated hepatocytes and CLP-induced sepsis models by modulating GPX4 expression (191). Therefore, targeting ferroptosis may offer a promising approach for treating septic liver injury.

#### 5.2.4 Sepsis-associated acute kidney injury

Sepsis often leads to AKI, which is marked by rapid deterioration in renal function. The incidence of SA-AKI has been increasing (192). Recent studies suggest that ferroptosis contributes significantly to the pathophysiology of AKI (193).

Fatty acid oxidation (FAO) is a primary energy source for proximal tubular epithelial cells. During ischemia, an imbalance between fatty acid intake and utilization impairs FAO due to the accumulation of free fatty acids. This disruption promotes lipid accumulation, enhances lipid peroxidation, and accelerates ROS production, thereby amplifying the inflammatory response and aggravating kidney injury.

Inhibition of NADPH oxidase, an antioxidant produced by mitochondria that increases NADPH concentrations, effectively reduces ferroptosis in renal cells (194). Ye et al. demonstrated that LOX inhibitors improve renal function, reduce proteinuria, and enhance creatinine clearance in LPS-induced AKI mice (195). These findings strongly suggest that targeting ferroptosis could be a viable strategy for treating SA-AKI.

#### 5.2.5 Sepsis-associated encephalopathy

SAE affects up to 70% of patients, with symptoms ranging from delusions and disorientation to coma (196, 197). The full pathophysiology of SAE remains unclear, but sepsis exacerbates SAE by promoting ferroptosis (198). Xie et al. showed that glutamate contributes to neuronal injury during SAE through ferroptosis (199). Thus, ferroptosis plays a crucial role in SAE development.

The hippocampus, critical for memory and learning, is particularly vulnerable to damage from inflammation and hypoxia (200). SAE-induced ferroptosis in the hippocampus is characterized by increased ROS, iron content, and MDA levels, alongside reduced GSH levels and altered expression of ferroptosis-related proteins (GPX4, ACSL4, and SLC7A11).

In CLP-induced mouse models, survival rates were reduced, and cognitive dysfunction, hippocampal damage, and mitochondrial injury were observed. However, in SAE mice, irisin alleviated neurological impairment, reduced hippocampal ferroptosis and microglial activation, and mitigated CLP-induced learning and memory deficits (201, 202).

Research in animal models of sepsis suggests that cytokines such as TNF-α and IL-6 cross the blood-brain barrier, disrupting cerebral microcirculation and leading to oxidative stress, mitochondrial damage, and cell death (199). Therefore, ferroptosis is a key contributor to SAE pathogenesis.

#### 5.2.6 Sepsis-associated immunosuppression

The role of ferroptosis in sepsis-related immunosuppression is gaining recognition, with immune cell depletion and immune dysfunction being prominent effects. However, further research is needed to clarify the specific pathways and mechanisms through which ferroptosis regulates immune suppression.

### 5.3 Other critical illness

#### 5.3.1 ARDS/ALI

ARDS and ALI are characterized by diffuse interstitial and alveolar edema, which can lead to hypoxic respiratory insufficiency or failure. These conditions can be triggered by both direct (intrapulmonary) and indirect (extrapulmonary) factors. Ferroptosis significantly impacts the onset and progression of ALI (203).

Mice with ALI induced by oleic acid (OA) or LPS exhibited iron overload, reduced GSH, MDA, and 4HNE levels, and downregulated ferritin, SLC7A11, and GPX4 expression (204, 205). Downregulation of CircEXOC5 mitigated lung injury by reducing ROS levels, downregulating GPX4, and upregulating ACSL4, thus suppressing ferroptosis (206).

Purearin has been shown to reduce epithelial damage by lowering iron concentrations in lung epithelial cells and suppressing GPX4 and GSH expression, thereby inhibiting ferroptosis (207). Additionally, electroacupuncture (EA) stimulation at Zusanli (ST36) prevents ferroptosis in alveolar epithelial cells by activating the α7 nicotinic acetylcholine receptor (α7nAchR), mitigating LPS-induced ARDS/ALI (208). These findings suggest that targeted suppression of ferroptosis in the lungs may benefit ARDS/ALI patients.

#### 5.3.2 AKI

AKI is a prevalent clinical condition, affecting 11.6% of individuals in China (209). Approximately 700,000 adult hospitalizations annually result in death from AKI (210). Ferroptosis plays a pivotal role in the development of AKI (211). Among the various causes of AKI, tubular cells, particularly the metabolically active and energy-dense proximal tubular cells, are highly susceptible to ferroptosis (212). Therefore, ferroptosis-induced proximal tubular injury may be a key mechanism in the pathogenesis of AKI.

In the context of rhabdomyolysis, myocyte rupture releases significant amounts of myoglobin, which is subsequently broken down into iron and heme. These byproducts damage tubular epithelial cells through lipid peroxidation, eventually leading to ferroptosis. The transcriptional regulator YAP mediates the upregulation of ACSL4 in skeletal muscle cells, contributing to ferroptosis and promoting the progression of rhabdomyolysis following exercise-induced heatstroke. Targeted suppression of ACSL4 and ferroptosis may, therefore, offer a potential therapeutic strategy to mitigate rhabdomyolysis-induced AKI (213). Furthermore, Wang et al. demonstrated that ACSL4 deficiency protects against AKI mediated by ferroptosis (214).

Cisplatin, a first-generation platinum chemotherapy drug, is associated with nephrotoxicity, which remains its primary side effect. Both a single high dose and repeated low doses can lead to AKI. To prevent AKI progression, Zhao et al. administered iron sucrose to a cisplatin-induced AKI mouse model (215). Their findings indicated that iron supplementation upregulated ferritin and iron transporters. Additionally, activation of vitamin D receptors partially inhibited ferroptosis through GPX4 trans-regulation, thus alleviating cisplatin-induced AKI (216).

In GPX4 knockout mice, there is a notable increase in both the incidence and mortality of spontaneous AKI (20). Legumain, a conserved asparagine endopeptidase, stabilizes GPX4 in its absence, thereby reducing tubular cell ferroptosis (217). Moreover, HO-1 significantly inhibits ferroptosis in renal proximal tubular cells (218). As such, targeting ferroptosis holds promise for improving therapeutic outcomes in AKI.

#### 5.3.3 Acute liver injury

Acute liver injury is characterized by a sudden decline in hepatic function, typically without prior signs of liver disease. It is caused by hepatotoxic agents such as alcohol, narcotics, IRIs, or viral hepatitis (219). Numerous studies have explored the mechanism of ferroptosis induced by acetaminophen (also known as N-acetyl-p-aminophenol, or APAP), a widely used nonsteroidal anti-inflammatory drug (220). Intracellular GSH levels are critical for the activation of ferroptosis, with APAP overdose leading to a significant depletion of GSH (221).

The mechanisms underlying APAP-induced liver injury have been linked to iron accumulation and lipid peroxidation. Yamada et al. identified ferroptosis, marked by increased lipid peroxides derived from n-6 PUFAs, as a key factor in APAP-induced acute liver injury in a mouse model (222). Moreover, mice deficient in GPX4 showed that α-tocopherol could inhibit excessive lipid peroxidation (223).

Recent research underscores the pivotal role of mitochondria in ferroptosis development (224). In animal models of APAP-induced acute liver injury, ferroptosis inhibitors, such as UAMC-3203 and VDAC1 oligomerization inhibitor VBIT-12, were shown to preserve mitochondrial function and reduce ferroptosis (225). These findings suggest that ferroptosis may serve as a promising therapeutic target for treating acute liver injury.

#### 5.3.4 Stroke

Stroke, encompassing both hemorrhagic and ischemic subtypes, has been linked to ferroptosis (226). Following severe hypoxic-ischemic brain injury, regions such as the thalamus, subcortical white matter, periventricular areas, and basal ganglia exhibit significant iron accumulation (227).

In ischemic stroke models, a reduction in GSH levels and GPX4 activity is observed, along with increased lipid peroxidation in neuronal cells (228). Lu et al. demonstrated that lncRNA PVT1, through miR-214-mediated modulation of TFR1 and p53 expression, regulates ferroptosis in acute ischemic stroke (229).

Ferroptosis also contributes to neuronal death after hemorrhagic stroke (230). In a hemorrhagic stroke model, GPX4 levels significantly decreased within 24 hours post-intracerebral hemorrhage. However, a single dose of selenium enhanced GPX4 expression, mitigating inflammation, oxidative stress, blood-brain barrier disruption, cerebral edema, and neuronal dysfunction (231). Therefore, ferroptosis modulators represent potential pharmacological targets for stroke therapy.

#### 5.3.5 Traumatic brain injury

TBI is a major cause of head injuries, fatalities, and disabilities due to accidents such as falls and crashes (232). Ferroptosis contributes to neurological impairment and neuronal death following TBI (233). Pathophysiological mechanisms include iron accumulation, disrupted iron metabolism, overexpression of ferroptosis-related genes, reduced GPX activity, and increased ROS levels, all of which are linked to TBI progression (234).

Ruxolitinib has demonstrated neuroprotective effects in animal models of TBI by inhibiting the JAK-STAT pathway, thereby preventing iron accumulation, neurotoxicity, brain edema, and lesion expansion. This treatment also improves motor deficits, cognitive dysfunction, and anxiety-like behaviors (235). Moreover, ruxolitinib reverses the decrease in GPX4 levels and the increase in TfR1, cyclooxygenase-2 (COX2), and 4-HNE observed in the acute phase of TBI.

Liang et al. reported that peroxisome proliferator-activated receptor γ (PPARγ) expression was reduced in neurons undergoing ferroptosis following TBI (236). However, pioglitazone, a PPARγ agonist, reversed this reduction, alleviating neuronal ferroptosis by reducing MDA and COX2 levels both *in vitro* and *in vivo*. Furthermore, pioglitazone reduced neuronal loss, lesion size, and neurological severity scores (NSS) in TBI mice.

In summary, inhibiting ferroptosis offers a promising therapeutic strategy for neuroprotection in TBI. A deeper understanding of the mechanisms underlying ferroptosis-targeting treatments will pave the way for novel therapeutic approaches for TBI patients. This could significantly improve clinical outcomes and establish a new theoretical framework for TBI management.

## 6 Therapeutic approaches targeting ferroptosis in critical care diseases

Ferroptosis is a key target for therapeutic interventions aimed at both treating and preventing severe illnesses, as it plays a role in the progression of various critical conditions. This paper reviews several drugs and compounds known to inhibit ferroptosis, exploring their potential applications in a range of serious diseases (see **Supplementary Table 1**).

### 6.1 IRI

#### 6.1.1 Cardiac IRI

Feng et al. utilized a mouse cardiac I/R model to demonstrate that early administration of Liproxstatin-1 (Lip-1) during reperfusion increased GPX4 levels in mitochondria, thereby reducing ischemic infarct size and improving mitochondrial function and morphology (237). Both deferiprone (DFP) and deferoxamine (DFO) mitigate myocardial IRI by inhibiting ferroptosis (149, 150). Additionally, 2,2-Bipyridine and MitoFerroGreen have been shown to target mitochondrial iron reduction pharmacologically, offering protection against cardiac IRI in mice (238, 239).

Compound 968 has been shown to reduce myocardial IRI in ex vivo studies by limiting glutamine availability (150). Moreover, by inhibiting p53, USP22-mediated deubiquitination prevents ferroptosis, leading to the overexpression of SLC7A11 and a decrease in ROS generation, which ultimately reduces myocardial IRI (240). Baicalin exerts its protective effects by inactivating ACSL4, thus resisting ferroptosis and preventing myocardial IRI (56). Other compounds, such as xanthohumol, naringenin, and resveratrol, have been shown to protect against I/R-induced heart ferroptosis by modulating GPX4 levels (241–243).

#### 6.1.2 Hepatic IRI

The first-generation ferroptosis inhibitor, ferrostatin-1 (Fer-1), functions as a synthetic antioxidant, preventing membrane lipid damage and inhibiting cell death (11). In animal models, Fer-1 and α-tocopherol have been demonstrated to reduce inflammatory responses and ameliorate hepatic IRI (20, 156). Li et al. showed that inhibiting ferroptosis with Lip-1 significantly reduced myeloperoxidase (MPO) activity and alleviated liver histopathological damage (146).

#### 6.1.3 Renal IRI

In a renal IRI model, administration of Lip-1 improved ferroptosis inhibition (241). Zhao et al. found that renal tubular epithelial cells undergoing IRI-induced AKI exhibited ferroptosis, while XJB-5-131 ameliorated IRI-induced AKI by inhibiting ferroptosis (244).

#### 6.1.4 Cerebral IRI

Lip-1 also protected mice from brain IRI by modulating iron-related gene and protein activity (234). The antioxidant N-acetylcysteine (NAC) prevented ferroptosis induced by cysteine depletion and System Xc⁻-inhibition by enhancing cysteine bioavailability (245, 246). Furthermore, NAC inhibits heme-induced ferroptosis in brain cells by opposing the toxic lipids produced through arachidonate 5-lipoxygenase (ALOX5) activity and cooperating with prostaglandin E2 (247).

#### 6.1.5 Bowel IRI

DFO has been shown to prevent lipid peroxidation induced by intestinal I/R and restores reduced GPX4 activity (248). Nrf2 agonists, such as dimethyl fumarate and signal transducer and activator of transcription 3 (STAT3), mitigate ferroptosis in intestinal IRI-ALI by increasing the mRNA expression of FTH1 and GPX4, reducing lipid peroxidation, and preserving GSH levels through upregulation of SLC7A11. This decreases lipid ROS and intracellular ROS levels (249, 250).

#### 6.1.6 Pulmonary IRI

Fer-1 and Lip-1 alleviate pulmonary edema, atelectasis, necrosis, inflammation, and fibrosis in I/R mice, exacerbated by iron or Erastin treatment through tail vein injection (170, 249). However, while ferroptosis inhibitors have shown lung protection in animal models, they have not yet been translated into clinical practice, requiring further investigation into their regulatory mechanisms.

### 6.2 Sepsis

#### 6.2.1 Sepsis-induced cardiac injury

The α2 adrenergic agonist dexmedetomidine (Dex) reduces ferroptosis by activating the SLC7A11-GPX4 signaling pathway. Clinical studies indicate that Dex mitigates myocardial injury in sepsis, although its precise mechanism remains unclear (251). Similarly, DFP and DFO attenuate sepsis-induced cardiac injury by inhibiting ferroptosis (252).

#### 6.2.2 SALI/ARDS

In patients with SALI, hydrogen sulfide reduces ferroptosis and blocks mTOR signaling (253). Additionally, the RNA-binding protein AUF1 contributes to improved SALI outcomes by preventing ferroptosis via the Nrf2 pathway (254). AUF1 suppression exacerbates lung injury by inducing ferroptosis and significantly reduces survival in a CLP-induced SALI model (207).

Intraperitoneal injection of recombinant cold-induced RNA-binding protein (CIRP) downregulates GPX4 expression and elevates lipid ROS levels in lung tissue. This effect is mitigated by Fer-1. Ferroptosis and lung damage are less prevalent in CIRP mutant cells in CLP-induced SALI models, and the CIRP inhibitor C23 further reduces ferroptosis and SALI severity *in vivo* (255).

#### 6.2.3 Sepsis-related liver injury

Serum levels of the specialized protein MFG-E8, which inhibits ferroptosis, are lower in septic patients compared to healthy individuals (256). Intraperitoneal administration of recombinant MFG-E8 in mice improves liver recovery, reduces oxidative stress and ferroptosis in liver tissue, and enhances survival rates in septic mice.

#### 6.2.4 SA-AKI

Antioxidants that neutralize free radicals, such as vitamin E and phenolic compounds, have been shown to enhance ferroptosis and improve renal function (257). Maresin conjugates in tissue regeneration-1 (MCTR1) mitigate SA-AKI by modulating Nrf2 expression, thereby further preventing renal ferroptosis (258).

#### 6.2.5 SAE

In SAE, the ferroptosis inhibitor Fer-1 prevents neuronal death and reduces glutamate toxicity (259). Animal studies have also shown that Fer-1 significantly improves survival rates in SAE mice and alleviates cognitive dysfunction by inhibiting ferroptosis (260). In parallel, the mTOR inhibitor rapamycin reduces inflammatory responses, mitigates SAE, and improves cognitive deficits in septic mice by activating autophagy and suppressing pyroptosis (261, 262).

### 6.3 Other critical illness

#### 6.3.1 ARDS/ALI

*In vitro* and *in vivo* studies have demonstrated that panaxydol (PX), a polyacetylene compound from ginseng root, dose-dependently increases GSH and GPX4 expression while significantly reducing Fe^2+^ accumulation. These effects lead to substantial improvements in lung histopathology in lipopolysaccharide-induced injury (183).

Artesunate (AS) enhances the anti-apoptotic pathways of Nrf2 and HO-1 while activating the mTOR/AKT signaling pathway, reducing lung tissue damage and neutrophil infiltration (263, 264). Certain metabolites, including oxgonolone and itaconic acid, also protect against LPS-induced ALI by activating the Nrf2 pathway (204, 265).

#### 6.3.2 AKI

Skouta et al. initially confirmed the ferroptosis-inhibiting effects of hepostatin-1 in a rhabdomyolysis-induced AKI model (266). Fer-1 significantly reduces creatinine and blood urea nitrogen levels, mitigating kidney damage in cisplatin-induced AKI and nephrotoxic folate-induced AKI models (216, 267). FG-4592 prevents ferroptosis by activating Nrf2, protecting against folate-induced kidney damage (268). Paricalcitol inhibits cisplatin-induced AKI by modulating GPX4’s antioxidant properties through vitamin D receptor activation (216).

Quercetin inhibits ferroptosis by increasing GSH levels and reducing both MDA and lipid ROS levels (269). Nuciferine directly reduces ferroptosis by limiting iron accumulation, reducing oxidative stress, and minimizing lipid peroxidation, all in a GPX4-dependent manner (270). Tocilizumab and small-molecule compounds from traditional Chinese medicine (e.g., nobiletin and tectorigenin) also inhibit fibrotic progression and ferroptosis (271–273).

#### 6.3.3 Acute liver injury

Lőrincz et al. demonstrated that Fer-1 offers limited protection against APAP-induced ferroptosis in primary murine hepatocytes (274). According to Schnellmann et al., DFO’s intracellular iron chelation reduces liver injury caused by APAP (275).

The antioxidant protein sestrin2 (Sesn2) plays a crucial role in responding to various stressors and maintaining cellular homeostasis (276). Cells expressing Sesn2 exhibit resistance to ferroptosis, ROS generation, and GSH depletion induced by erastin. *In vivo*, adenovirus-mediated Sesn2 expression completely mitigated the elevated serum ALT/AST levels and histological changes in animals pretreated with phenylhydrazine (277).

Glycyrrhizic acid, an inhibitor of HMGB1, can suppress ferroptosis by mitigating oxidative stress, thus reducing liver injury (278). Similarly, promethazine alleviates acute liver failure induced by LPS and d-galactosamine (GalN) by inhibiting lipid peroxidation and reducing cell death (279).

#### 6.3.4 Stroke

In the context of stroke, Tuo et al. demonstrated that tau-mediated iron export alleviated ferroptosis-induced damage in gerbils following ischemic stroke. Carvacrol further inhibited ferroptosis by enhancing GPX4 levels (280, 281). DFO effectively blocked brain injury induced by MCAO, although its short half-life limits its efficacy, requiring sustained administration for cerebral protection (282). Compound 2-(1-(4-(4-methylpiperazin-1-yl)phenyl)ethyl)-10H-phenothiazine (51) also demonstrated efficacy in preventing ferroptosis in an MCAO ischemic stroke model (283).

Furthermore, Fer-1 significantly mitigated secondary brain injury following intracerebral hemorrhage (284). When administered via lateral ventricle injection, Fer-1 reduced lesion volume, neurodegeneration, and iron deposition, ultimately improving the long-term cognitive outcomes of affected animals (234).

#### 6.3.5 TBI

In TBI, overexpression of Lip-1 in a mouse model improved cognitive function by reducing brain lesion size and neurodegeneration (285). The neuroprotective effects of Lip-1 are attributed to its ability to lower iron and lipid peroxidation levels and restore glutathione in the injured brain tissue. Polydatin has been shown to reverse the deposition of free iron ions/Fe²⁺, elevate MDA levels, decrease GPX4 activity in the injured area, and protect neurons in TBI models (286).

Tetrandrine (Tet) treatment improved neurological scores in TBI mice, reduced brain contusion and edema, and increased expression of GPX4, GSH, SLC7A11, and FTH, while decreasing MDA levels (287). Melatonin also mitigates lipid peroxidation via the circPtpn14/miR-351-5p/5-LOX signaling cascade, reducing iron deposition and neurodegeneration, ultimately improving neurological function after TBI (288, 289).

## 7 Clinical application of ferroptosis

Fer-1 has shown protective effects against doxorubicin-induced cardiac injury without altering iron levels, preventing cell death in cultured cardiomyocytes (72, 290), and reducing mitochondrial damage. Additionally, Fer-1 significantly alleviates cardiac injury, inflammation, and survival time in LPS-treated mice (252). Fer-1 has also been demonstrated to inhibit iron deposition in various acute kidney injury models, exerting renoprotective effects (193). Lip-1, a spiroquinoxaline amine derivative identified through high-throughput screening in Gpx4^−/−^ cells (20), has been shown to significantly reduce palmitate-induced cardiac injury (291). In both unilateral ureteral obstruction (UUO) and radiation-induced lung fibrosis models, Lip-1 administration improved ferroptosis and fibrosis (292, 293) outcomes. MitoTEMPO, a mitochondria-targeted antioxidant, prevented ferroptosis-induced cardiac injury by inhibiting lipid peroxidation in murine hearts (72).

Ferroptosis, an iron-dependent form of programmed cell death, can be inhibited by iron chelators (11). Dexrazoxane, a cyclic ethylenediaminetetraacetic acid derivative, easily crosses cell membranes and accumulates in the mitochondria of cardiomyocytes, where it chelates intracellular free iron (294, 295). Similarly, DFO reduces ferroptosis and fibrosis in CKD rat models (296). While iron chelators protect various tissues and organs from injury, their protective effects may not solely result from ferroptosis inhibition.

Rapamycin reduces TfR1 expression, lowering intracellular iron levels, and activates the IRP1/2 system to compensate for the downregulation of iron transporters, thereby shielding cardiomyocytes from iron overload and ferroptosis (297, 298). Zinc protoporphyrin IX, a competitive inhibitor of HO-1, prevents doxorubicin-induced iron accumulation and ferroptosis in the murine heart by blocking heme degradation and limiting free iron release (72). Additionally, atorvastatin alleviates isoproterenol-induced cardiac dysfunction and remodeling in mice by inhibiting ferritin autophagy-mediated ferroptosis (299). Puerarin protects cultured cardiomyocytes from ferroptosis induced by both erastin and isoproterenol (300). Cyanidin-3 glucoside (CG3), an anthocyanin, mitigates ferroptosis by reducing Fe^2+^ content through TFR1 and FTH1 modulation, which in turn reduces myocardial IRI (301).

## 8 Discussion and prospect

As research progresses, ferroptosis is increasingly recognized for its critical role in various diseases, offering new perspectives for the treatment of related disorders. The involvement of ferroptosis in IRI and sepsis-induced tissue damage varies across organs, influenced by differences in metabolic activity, iron homeostasis, antioxidant defenses, and cellular sensitivity to oxidative stress. Understanding these variations clarifies why certain tissues are more vulnerable under pathological conditions.

Organs such as the heart, brain, and kidneys, which have high metabolic rates and oxygen demands, are particularly susceptible to severe oxidative stress during IRI and sepsis. Reperfusion after ischemia triggers increased ROS production. For example, the brain’s high PUFA content makes it especially vulnerable to lipid peroxidation, a key step in ferroptosis (162). In contrast, tissues with lower metabolic rates generate fewer ROS and are less prone to ferroptosis.

Iron metabolism varies across tissues, influencing susceptibility to ferroptosis. The liver, a primary iron storage site, has elevated iron levels that promote ROS production, increasing ferroptosis risk during IRI or sepsis (277). Tissues with lower iron content, like skeletal muscle, exhibit reduced ferroptotic activity. Additionally, an organ’s antioxidant capacity is pivotal. The liver, equipped with robust antioxidants such as glutathione and superoxide dismutase (SOD), counters oxidative stress but becomes vulnerable to ferroptosis if these defenses are compromised during sepsis or IRI (277). Similarly, the kidney’s high susceptibility to ferroptosis arises when its protective mechanisms are overwhelmed, resulting in acute injury.

Lipid composition also plays a key role in ferroptosis susceptibility. The brain’s high PUFA content predisposes it to lipid peroxidation under stress, exacerbating damage during IRI and sepsis (227). Conversely, tissues like the pancreas, with distinct lipid profiles, show varied susceptibility. Organ-specific inflammatory responses during sepsis further influence ferroptosis development. The liver and kidneys, with significant immune cell infiltration, are particularly prone to ROS-induced damage and ferroptosis due to the pro-inflammatory environment (212).

Ferroptosis represents a unique form of cell death that interacts with other cell death pathways, offering potential for combination therapies in the future. Despite extensive research into the molecular mechanisms of ferroptosis, significant challenges remain, as the field is still emerging. For example, the molecular signaling pathways that regulate ferroptosis are not fully elucidated. The mechanisms behind p53’s bidirectional regulation in ferroptosis remain unclear. Furthermore, while iron is crucial for ferroptosis, it is unknown whether other metal ions can replace it. What are the downstream genes regulated by iron metabolism, and what are the specific molecular mechanisms involved? Moreover, current techniques cannot detect ferroptosis *in vivo* through specific biomarkers. Identifying biomarkers that reflect ferroptosis and disease severity would significantly enhance clinical diagnostics. Future research could focus on discovering ferroptosis biomarkers through transcriptional or metabolomic approaches. Additionally, the clinical efficacy of ferroptosis inhibitors remains uncertain, presenting a major research gap. Although some studies have explored clinically used drugs in animal models or specific cell types, research on clinical safety and efficacy is sparse. Comprehensive studies are needed to define the therapeutic potential of ferroptosis inducers and inhibitors, including optimal timing, dosages, administration routes, and treatment durations. Further preclinical and clinical research is crucial to assess ferroptosis’s impact in humans and facilitate the development of targeted therapies for human diseases.

In conclusion, investigating ferroptosis, a recently identified cell death pathway, may lead to groundbreaking advancements in critical care and the treatment of severe diseases. Key questions remain, such as how ferroptosis is regulated in various critical conditions and how best to modulate it. Answers to these questions will provide a foundation for halting disease progression and identifying effective therapeutic targets.
