# Supplementary material for: Deciphering ferroptosis in critical care: mechanisms, consequences, and therapeutic opportunities
Source: Front Immunol. 2024 Dec 16;15:1511015. doi: 10.3389/fimmu.2024.1511015 (PMC11682965; doi:10.3389/fimmu.2024.1511015)
Supplement: Supplementary file 1 [file Table1.docx]

**TABLE 1**: Effect of ferroptosis-related drugs on critical illness and its underlying mechanism.

| Critical illness | Drugs | Ferroptosis-related targets | Study subject | Mechanism | Reference |
| --- | --- | --- | --- | --- | --- |
| Cardiac IRI | ATF3 | SLC7A11 | NMCs | Reduced Fe^2+^, ROS, MDA levels and cell death | (1) |
|  | USP7 Inhibitor | TFRC | SD rats | Reduce ferroptosis by reducing iron content and lipid peroxidation | (2) |
|  | Lip-1 | GPX4 | Isolated perfused mice | Lip-1 was able to decrease the expression of voltage-dependent anion channel protein 1 in the outer mitochondrial membrane, further increase the content of antioxidant GPX4 in mitochondria, and reduce ROS production in mitochondria | (3) |
|  | DFP | Ferrous ion | SD rats | DFP removes intracellular iron, iron in the reticuloendothelial system, transferrin bound iron, and iron stored as ferritin and hemosiderin | (4) |
|  | DFO | Ferrous ion | MEFs | Continuous high-dose infusion of DFO can significantly reduce cardiotoxicity caused by iron overload, relatively quickly improve ventricular function, reduce iron overload-related complications, and improve overall survival and quality of life of patients | (5) |
|  | 2,2-Bipyridine | Mitochondrial iron | DIC mice | 2,2-Bipyridine targets mitochondrial iron reduction, reduces iron accumulation, and avoids iron overload. | (6) |
|  | MitoFerroGreen | Mitochondrial iron | C57/BL6 mice, Patients | MitoFerroGreen decreases Fe^2+^ in mitochondria | (7) |
|  | Compound 968 | Glutamine | MEFs | Compound 968 is a cell-permeable small-molecule glutaminolysis inhibitor that attenuates myocardial ischemia-reperfusion injury ex vivo by limiting glutaminolysis | (5) |
|  | USP22 deubiquitination | P53 | SD rats | Inhibition of P53 negatively regulates ferroptosis by increasing expression levels of SLC7A11 and reducing ROS production | (8) |
|  | Baicalin | ACSL4 | H9c2 cells | Baicalin is a class of lipophilic flavonoid glycosides with antioxidant effects, which can inactivate ACSL4 to resist ferroptosis to prevent myocardial IRI | (9) |
|  | Xanthohumol | GPX4 | Male CD1 mice, RAW264.7 cells, NRK-52E cells | Xanthohumol, a flavonoid, exerts protective effects against I/R-induced cardiac ferroptosis in isolated hearts by modulating GPX4 protein levels | (10) |
|  | Resveratrol | GPX4 | SD rats, H9C2 cells | Resveratrol can increase the expression of GPX4, reduce oxidative stress, inhibit ferroptosis, and ameliorate myocardial IRI | (11) |
| Hepatic IRI | α-tocopherol | Lipid peroxidation | C57/BL6 mice | α-tocopherol, as an inhibitor of lipid peroxidation, can improve the inflammatory response and reduce hepatic IRI. | (12) |
|  | Lip-1 | System Xc^-^ | Male C57BL/6 mice, Caco-2 cells | Lip-1 significantly decreased MPO activity and reduced histological damage in the liver | (13) |
| Renal IRI | ALR | ROS | HK‐2 cells | ALR reduces ROS, mitochondrial damage, inhibits ferroptosis in renal I/R and impacts renal injury | (14) |
|  | Lip-1 | System Xc^-^ | Male CD1 mice, RAW264.7 cells, NRK-52E cells | Lip-1 inhibits lipid peroxidation by scavenging ROS, thereby inhibiting the ferroptosis caused by inactivation of the cystine transporter System Xc^-^ and GPX4. | (10) |
|  | XJB-5-131 | ROS | TECs | XJB-5-131 has dual antioxidant effects, which include the free radical scavenger TEMPO and semicgramicidins targeting mitochondria, which have high affinities for TECs and inhibit I/R induced TECs ferroptosis. | (15) |
| Cerebral IRI | Lip-1 | System Xc^-^, GPX4 | Male C57BL/6 mice | Lip-1 prevented brain IRI in mice by regulating expression levels of iron-related proteins and genes | (16) |
|  | NAC | System Xc^-^ | C57BL/6 and pregnant CD1 mice | NAC inhibits heme-induced brain cell ferroptosis by neutralizing toxic lipids produced by arachidonic acid-dependent ALOX5 activity and synergizing with prostaglandin E2 | (17) |
| Bowel IRI | IASPP | Nrf2 | C57BL/6J mice | IASPP inhibits intestinal I/R-induced ferroptosis by activating the Nrf2-HIF-1-TF signaling pathway. | (18) |
|  | Capsaicin ester | GPX4 | Male C57BL/6 mice | Capsaicin ester activates member 1 of the transient receptor-potential cation channel subfamily V and enhances GPX4 expression, thereby inhibiting cellular ferroptosis | (19) |
|  | DFO | Lipid peroxidation；GPX4 | White Wistar rats | DFO helps prevent intestinal I/R-induced lipid peroxidation and reverses reduced GPX4 activity | (20) |
|  | Dimethyl fumarate | SLC7A11；GSH；Lipid peroxidation | C57BL/6J mice, MLE-12 cells | Increased mRNA expression of FTH1 and GPX4 by up-regulating levels of SLC7A11 and prevented GSH depletion and lipid peroxidation accumulation | (21) |
|  | STAT3 | SLC7A11；ROS | C57BL/6J mice, MLE-12 cells | Increased mRNA expression of FTH1 and GPX4 by up-regulating levels of SLC7A11 and decreased intracellular ROS and lipid ROS levels | (22) |
| Pulmonary IRI | Fer-1 | Lipid peroxidation | C57BL/6J mice | Fer-1 reduced lung edema, atelectasis, necrosis, inflammation, and fibrosis in I/R mice | (18) |
|  | Lip-1 | System Xc^-^, GPX4 | C57BL/6J mice, MLE-12 cells | Lip-1 reduced lung edema, atelectasis, necrosis, inflammation, and fibrosis in I/R mice | (21) |
| Sepsis-related cardiac injury | CLP | GPX4；GSH | C57BL/6J mice | CLP significantly decreased GPX4 and GSH levels and increased HO-1, TfR1, Caspase3, inducible nitric oxide synthase, and Fe^2+^ concentrations. | (23) |
|  | Dex | SLC7A11；GPX4 | C57BL/6 mice | Dex inhibits ferroptosis by activating the SLC7A11-GPX4 signaling pathway | (24) |
|  | DFP | Ferrous ion | SD rats | DFP removes intracellular iron, iron in the reticuloendothelial system, transferrin bound iron, and iron stored as ferritin and hemosiderin | (4) |
|  | DFO | Ferrous ion | C57BL/6 mice | DFO attenuates sepsis-associated cardiac injury by chelating iron ions, reducing free iron ions, mitigating iron overload, and blocking ferroptosis | (25) |
| SALI/ARDS | YAP1 | SLC7A11； FTH1；GPX4 | C57BL/6 mice, MLE-12 cells | In CLP-induced murine models, YAP1 elevated expression of GPX4, FTH1, and SLC7A11, reduced expression of NCOA4, and suppressed ferroptosis | (26) |
|  | American ginseng phenol | Nrf2；HO-1 | C57BL/6J mice, MLE-12 cells | American ginseng phenol could ameliorate ALI in sepsis by activating Nrf2 to promote HO-1 expression | (27) |
|  | Mucin | Nrf2；GPX4 | Male wild-type C57BL/6  Mice, THP-1cells | Mucins inhibit macrophage ferroptosis and alleviate SALI by up-regulating GPX4 expression through activation of Nrf2 related pathways | (28) |
|  | Itraconazole | Nrf2；GPX4 | Patients, Male C57BL/6J mice, MLE-12 cells | Itraconazole inhibits ferroptosis by upregulating GPX4 expression through activation of Nrf2 | (29) |
|  | Hydrogen sulfide | mTOR | C57BL/6J mice | Hydrogen sulfide attenuates ferroptosis and blocks mTOR signaling in SALI | (30) |
|  | AUF1 | Nrf2 | AECs | AUF1 inhibits ferroptosis through Nrf2 pathway to ameliorate SALI | (31) |
|  | CIRP inhibitor | GPX4 | C57BL/6 mice, RAW 264.7 cells | CIRP inhibitors could up-regulate GPX4 expression, reduce lipid ROS content in lung tissues, and decrease the ferroptosis and degree of lung injury in CLP-induced SALI model | (32) |
| Sepsis-related liver injury | YAP1 | NCOA4 | C57BL/6 mice | YAP1 protects against septic liver injury by reducing NCOA4 expression and inhibiting ferritin phagocytosis-mediated ferroptosis | (33) |
|  | Irisin | GPX4 | Patients, male C57BL/6J mice | Irisin can influence GPX4 expression and attenuate ferroptosis in LPS-treated hepatocytes and in a murine model of CLP sepsis | (34) |
|  | MFG-E8 | ROS | Male C57BL/6 mice, HK-2 cells | Intraperitoneal supplementation of recombinant MFG-E8 in mice ameliorates liver injury by inhibiting ferroptosis and oxidative stress in liver tissue cells | (35) |
| SA-AKI | NADPH oxidase | NADPH | C57BL/6 mice | NADPH oxidase is effective in reducing the ferroptosis of renal cells by increasing NADPH levels | (36) |
|  | LOX inhibitor | ROS | C57BL/6J mice | LOX inhibitors can improve renal function and reduce decreased creatinine clearance and increased proteinuria in LPS-induced AKI mice | (37) |
|  | Vitamin E | ROS | Male albino rats | Vitamin E captures oxygen free radicals, reduces oxidative stress, improves ferroptosis and improves kidney function | (38) |
|  | MCTR1 | Nrf2 | Male C57BL/6 mice, HK-2 cells | MCTR1 rescues SA-AKI by further inhibiting renal ferroptosis through modulating Nrf2 expression levels | (39) |
| SAE | Irisin | GPX4 | C57BL/6J mice, HT-22 cells | Irisin attenuated CLP-induced learning and memory dysfunction, neurological severity scores, and hippocampal ferroptosis and microglial activation in SAE mice by modulating GPX4 levels | (40) |
|  | Fer-1 | Glutamine | C57BL/6 mice | Fer-1 attenuates glutamate toxicity and inhibits neuronal death in SAEs | (41) |
|  | Rapamycin | mTOR | HUVECs, Human THP-1 cells, Kunming mice | Rapamycin attenuates inflammatory responses, sepsis-associated brain injury, and cognitive impairment in septic mice by inhibiting mTOR to activate autophagy and inhibit pyroptosis | (42,43) |
| ARDS/ALI | CircEXOC5 | ACSL4；GPX4；ROS | Patients | Downregulation of CircEXOC5 attenuates lung injury by decreasing ROS levels, downregulating GPX4 proteins, and upregulating ACSL4 expression to suppress ferroptosis | (44) |
|  | Puerarin | HO-1 | A549 cells | Puerarin prevented epithelial injury caused by ferroptosis by decreasing iron concentrations in lung epithelial cells and decreasing GPX4 and GSH expression and synthesis | (45) |
|  | EA | a7nAchR | Male C57BL/6 mice, MLE-12 cells | EA stimulation of Zusanli (ST36) attenuates LPS-induced ARDS/ALI by inhibiting LPS-induced alveolar epithelial cell ferroptosis via activation of a7nAchR | (46) |
|  | PX | GPX4；GSH | C57BL/6 mice, BEAS-2B cells | PX dose-dependently increased the expression of GPX4 and GSH, and decreasing the accumulation of Fe^2+^ significantly ameliorated lipopolysaccharide-induced pathological changes in lung tissues | (47) |
|  | AS | Nrf2；HO-1 | SD rats, Kunming  mice | AS up-regulates the expression of anti-apoptotic systems such as Nrf2 and HO-1 or activates the mTOR/PKB axis and attenuates neutrophil infiltration and pathological injury in lung tissues | (48,49) |
|  | Oxgonolone | Nrf2 | C57BL/6 mice, BEAS-2B cells | Oxprenorphone protects against LPS-mediated ALI models by inducing Nrf2 pathway activation | (50) |
|  | Itaconic acid | Nrf2 | New England white rabbits | Itaconic acid inhibits ferroptosis by inducing Nrf2 pathway activation | (51) |
| AKI | Iron | Ferritin | C57BL/6 mice | Iron supplementation can up-regulate ferritin and iron transporters, thereby preventing worsening AKI | (52) |
|  | Vitamin D receptor | GPX4 | C57BL/6 mice, HK-2 cells | Vitamin D receptors partially inhibits ferroptosis via GPX4 trans-regulation, thereby mitigating cisplatin-induced AKI | (53) |
|  | Fer-1 | Lipid peroxidation | C57BL/6 mice | Fer-1 can significantly reduce the levels of serum urea nitrogen and creatinine and relieve renal injury | (54) |
|  | FG-4592 | Nrf2 | Male C57BL/6 mice | FG-4592 plays a protective role in folate-induced renal injury by inhibiting ferroptosis through activation of Nrf2 | (55) |
|  | Paricalcitol | GPX4 | C57BL/6 mice, HK-2 cells | Paricalcitol inhibits cisplatin-induced AKI by modulating the antioxidant effects of GPX4 via activation of vitamin D receptors | (53) |
|  | Quercetin | GSH | NRK-52E Cells, HK-2 cells | Quercetin could inhibit ferroptosis by decreasing MDA and lipid ROS levels and increasing GSH levels | (56) |
|  | Nuciferine | GPX4 | HK-2 human proximal tubular epithelial cells, HEK293T HEK cells | Nuciferine directly inhibits ferroptosis in a GPX4-dependent manner by limiting iron accumulations, inhibiting oxidative stress, and preventing lipid peroxidation | (57) |
|  | Tocilizumab | IL-6 | Female C57BL/6J mice | Tocilizumab mimotope alleviates kidney injury and fibrosis by inhibiting IL-6 signaling and ferroptosis in UUO model | (58) |
|  | Nobiletin | ROS | Male C57BL/6J mice | Nobiletin diminished kidney fibrosis and the expression of EMT markers, ameliorated oxidative stress and ferroptosis-associated injury, and mitigated the inflammatory response in the kidneys of UUO mice | (59) |
|  | Tectorigenin | Smad3 | Male C57BL/6 mice, TECs | Tectorigenin protects against unilateral ureteral obstruction by inhibiting Smad3-mediated ferroptosis and fibrosis. | (60) |
| Acute liver injury | UAMC-3203 | Mitochondria | Male C57BL/6J mice, Hepa1-6 cells | UAMC-3203 reduces ferroptosis in APAP-induced ALI animal models by protecting mitochondrial function | (61) |
|  | VBIT-12 | Mitochondria | Male C57BL/6J mice, Hepa1-6 cells | VBIT-12 reduces ferroptosis in APAP-induced ALI animal models by protecting mitochondrial function | (61) |
|  | Fer-1 | Lipid peroxidation | HepG2 cells | Fer-1 provides modest protection against APAP-induced ferroptosis in primary murine hepatocytes by reducing lipid peroxide production | (62) |
|  | DFO | Ferrous ion | Male B6C3F1 mice | DFO chelating intracellular iron attenuates APAP-induced liver injury | (63) |
|  | Sesn2 | ROS；GSH | PHZ-induced mice, Ad-Sesn2  infected mice HepG2 cells, AML-12 cells, MEF cells | Cells expressing Sesn2 were resistant to erastin-induced ferroptosis, ROS formation, or GSH depletion | (64) |
|  | Glycyrrhizin | ROS | Male specific- pathogen-free C57BL/6 mice, L02 cells | Glycyrrhizic acid can suppress ferroptosis by inhibiting oxidative stress, thereby reducing the degree of liver injury | (65) |
|  | Promethazine | Lipid peroxidation | C57BL/6N male mice, H9C2 cells, NRK49F cells, HK2 cells, C2C12 cells, MDA-MB-231 cells, NRK52E cells, LLC-PK1 cells, Panc-1 cells, HUPECs | Promethazine modifies acute liver failure induced by LPS/d-GalN by inhibiting lipid peroxidation and reducing cell death | (66) |
| Stroke | Selenium | GPX4 | Male C57BL/6 mice, HT22 hippocampal neuroblast cells | A single dose of selenium could drive the expression of GPX4 and reduce neuronal dysfunction, brain edema, blood-brain barrier injury, oxidative stress, and inflammatory injury after intracerebral hemorrhage | (67) |
|  | Carvacrol | GPX4 | Gerbils | Carvacrol inhibited ferroptosis by increasing GPX4 | (68) |
|  | DFO | Ferrous ion | Male Sprague-Dawley rats | DFO effectively blocks MCAO-induced brain injury by chelating intracellular iron | (69) |
|  | Compound 2-(1-(4-(4-methylpiperazin-1-yl)phenyl)ethyl)-10H-phenothiazine (51) | Lipid peroxidation | SD rats, HT1080 cells | Compound 2-(1-(4-(4-methylpiperazin-1-yl)phenyl)ethyl)-10H-phenothiazine (51) prevented ferroptosis in MCAO ischemic stroke model by inhibiting lipid peroxidation | (70) |
|  | Fer-1 | Lipid peroxidation | Male C57BL/6 mice | Fer-1 reduced iron deposition, neurodegeneration, and lesion volume in injured tissue and improved long-term prognosis of cognitive impairment | (16) |
| TBI | Ruxolitinib | GPX4 | Adult male C57BL/6J mice | Ruxolitinib reversed the decline in GPX4 and increases in TfR1, COX2, and 4-HNE in the acute phase after TBI | (71) |
|  | Pioglitazone | PPAR3 | Adult male SPF/ICR mice | Pioglitazone attenuated neuronal ferroptosis by activating PPAR3 to downregulate COX2 expression and MDA levels in vivo and in vitro | (72) |
|  | Lip-1 | Lipid peroxidation；GSH | Male C57BL/6 mice | The neuroprotective effect of Lip-1 against TBI is associated with a decrease in iron content and lipid peroxides as well as recovery of GSH in brain injured tissues. | (73) |
|  | Polydatin | GPX4 | Neuro2A cells, Male C57BL/6 mice | Polydatin could reverse free iron ion/Fe^2+^ deposition, increase the content of MDA, decrease GPX4 activity in the injured tissue area, and protect neurons in TBI models | (74) |
|  | Tet | GPX4；GSH；SCL7A11 | Adult SPF C57BL/6 male mice | Tet treatment improved modified NSS after TBI, reduced brain contusion injury and brain edema, increased the expression of GPX4, GSH, SCL7A11 and FTH, and decreased MDA levels in TBI mice | (75) |
|  | Melatonin | CircPtpn14/miR-351-5p/5-LOX | Patients, Male C57BL/6 mice, bEnd3 cells | Melatonin attenuates lipid peroxidation through circPtpn14/miR-351-5p/5-LOX signaling, thereby reducing iron deposition and neurodegeneration after TBI thereby improving neurological dysfunction after TBI | (76) |

Abbreviations: IRI, Ischemia-reperfusion injury; ATF3, Activating transcription factor 3; SLC7A11, Solute carrier family 7 member 11; ROS, Reactive oxygen species; MDA, Malondialdehyde; USP7, Ubiquitin-specific proteinase 7; TFRC, Transferrin receptor complexes; Lip-1, Liproxstatin-1; GPX4, Glutathione peroxidase 4; DFP, Deferiprone; DFO, Deferoxamine; USP22, Ubiquitin-specific proteinase 22; P53, Protein 53; ACSL4, Acyl-CoA synthetase long-chain family member 4; Fer-1, Ferrostatin-1; MPO, Myeloperoxidase; ALR, Augmenter of liver regeneration; TEMPO, 2,2,6,6-tetramethylpiperidin-1-oxyl; TEC, tubular epithelial cells; NAC, N-acetylcysteine; ALOX5, Arachidonate 5-lipoxygenase; IASPP, Inhibitor of apoptosis stimulating protein of p53, Nrf2, Nuclear factor erythroid 2-related factor 2; I/R, Ischemia/Reperfusion; HIF, Hypoxia-inducible factor; TF,Transferrin; GSH, Glutathione; FTH1, Ferritin heavy chain-1; STAT3, Signal transducer and activator of transcription 3; CLP, Cecal ligation and puncture; HO-1, Heme oxygenase 1; TfR1, Transferrin receptor 1; Dex, Dexmedetomidine; SALI, Sepsis-induced acute lung injury; ARDS, Acute respiratory distress syndrome; YAP1, Yes1 associated transcriptional regulator; NCOA4, Nuclear receptor coactivator 4; ALI, Acute lung injury; mTOR, mechanistic target of rapamycin; AUF1, AU-rich element RNA binding factor 1; CIRP, Cold-induced RNA binding protein; LPS, Lipopolysaccharide; MFG-E8, Epidermal growth factor 8; SA-AKI, Sepsis associated acute kidney injury; NADPH, Nicotinamide adenine dinucleotide phosphate; LOX, Lipid oxidases; AKI, Acute kidney injury; MCTR1, Maresin conjugates in tissue regeneration-1; SAE, Sepsis-associated encephalopathy; EA, Electroacupuncture; PX, Panaxydol; PKB, Protein Kinase B; FG-4592, Roxadustat; IL-6, Interleukin-6; UUO, Unilateral ureteric obstruction; EMT, Epithelial-mesenchymal transition; APAP, Acetaminophen; Sesn2, Sestrin2; GalN, galactosamine; MCAO, Middle cerebral artery occlusion; TBI, Traumatic head injury; COX2, Cyclooxygenase-2; 4-HNE, 4-hydroxynonenal; PPAR3, Peroxisome proliferator-activated receptor 3; Tet, Tetrandrine; NSS, Neurological severity score.

**REFERENCES**

1. Liu H, Mo H, Yang C, Mei X, Song X, Lu W, et al. A novel function of ATF3 in suppression of ferroptosis in mouse heart suffered ischemia/reperfusion. *Free Radic Biol Med* (2022) **189**: 122-35. doi:10.1016/j.freeradbiomed.2022.07.006

2. Tang LJ, Zhou YJ, Xiong XM, Li NS, Zhang JJ, Luo XJ, et al. Ubiquitin-specific protease 7 promotes ferroptosis via activation of the p53/TfR1 pathway in the rat hearts after ischemia/reperfusion. *Free Radic Biol Med* (2021) **162**: 339-52. doi:10.1016/j.freeradbiomed.2020.10.307

3. Feng Y, Madungwe NB, Imam AA, Tombo N, Bopassa JC. Liproxstatin-1 protects the mouse myocardium against ischemia/reperfusion injury by decreasing VDAC1 levels and restoring GPX4 levels. *Biochem Biophys Res Commun* (2019) **520**: 606-11. doi:10.1016/j.bbrc.2019.10.006

4. Tang LJ, Luo XJ, Tu H, Chen H, Xiong XM, Li NS, et al. Ferroptosis occurs in phase of reperfusion but not ischemia in rat heart following ischemia or ischemia/reperfusion. *Naunyn Schmiedebergs Arch Pharmacol* (2021) **394**: 401-10. doi:10.1007/s00210-020-01932-z

5. Gao M, Monian P, Quadri N, Ramasamy R, Jiang X. Glutaminolysis and Transferrin Regulate Ferroptosis. *Mol Cell* (2015) **59**: 298-308. doi:10.1016/j.molcel.2015.06.011

6. Tadokoro T, Ikeda M, Ide T, Deguchi H, Ikeda S, Okabe K, et al. Mitochondria-dependent ferroptosis plays a pivotal role in doxorubicin cardiotoxicity. *Jci Insight* (2020) **5**. doi:10.1172/jci.insight.132747

7. Chang HC, Wu R, Shang M, Sato T, Chen C, Shapiro JS, et al. Reduction in mitochondrial iron alleviates cardiac damage during injury. *Embo Mol Med* (2016) **8**: 247-67. doi:10.15252/emmm.201505748

8. Ma S, Sun L, Wu W, Wu J, Sun Z, Ren J. USP22 Protects Against Myocardial Ischemia-Reperfusion Injury via the SIRT1-p53/SLC7A11-Dependent Inhibition of Ferroptosis-Induced Cardiomyocyte Death. *Front Physiol* (2020) **11**: 551318. doi:10.3389/fphys.2020.551318

9. Fan Z, Cai L, Wang S, Wang J, Chen B. Baicalin Prevents Myocardial Ischemia/Reperfusion Injury Through Inhibiting ACSL4 Mediated Ferroptosis. *Front Pharmacol* (2021) **12**: 628988. doi:10.3389/fphar.2021.628988

10. Zhou L, Xue X, Hou Q, Dai C. Targeting Ferroptosis Attenuates Interstitial Inflammation and Kidney Fibrosis. *Kidney Dis (Basel)* (2022) **8**: 57-71. doi:10.1159/000517723

11. Wang C, Zhu L, Yuan W, Sun L, Xia Z, Zhang Z, et al. Diabetes aggravates myocardial ischaemia reperfusion injury via activating Nox2-related programmed cell death in an AMPK-dependent manner. *J Cell Mol Med* (2020) **24**: 6670-79. doi:10.1111/jcmm.15318

12. Yamada N, Karasawa T, Kimura H, Watanabe S, Komada T, Kamata R, et al. Ferroptosis driven by radical oxidation of n-6 polyunsaturated fatty acids mediates acetaminophen-induced acute liver failure. *Cell Death Dis* (2020) **11**: 144. doi:10.1038/s41419-020-2334-2

13. Li Y, Feng D, Wang Z, Zhao Y, Sun R, Tian D, et al. Ischemia-induced ACSL4 activation contributes to ferroptosis-mediated tissue injury in intestinal ischemia/reperfusion. *Cell Death Differ* (2019) **26**: 2284-99. doi:10.1038/s41418-019-0299-4

14. Huang LL, Liao XH, Sun H, Jiang X, Liu Q, Zhang L. Augmenter of liver regeneration protects the kidney from ischaemia-reperfusion injury in ferroptosis. *J Cell Mol Med* (2019) **23**: 4153-64. doi:10.1111/jcmm.14302

15. Zhao Z, Wu J, Xu H, Zhou C, Han B, Zhu H, et al. XJB-5-131 inhibited ferroptosis in tubular epithelial cells after ischemia-reperfusion injury. *Cell Death Dis* (2020) **11**: 629. doi:10.1038/s41419-020-02871-6

16. Xie BS, Wang YQ, Lin Y, Mao Q, Feng JF, Gao GY, et al. Inhibition of ferroptosis attenuates tissue damage and improves long-term outcomes after traumatic brain injury in mice. *Cns Neurosci Ther* (2019) **25**: 465-75. doi:10.1111/cns.13069

17. Karuppagounder SS, Alin L, Chen Y, Brand D, Bourassa MW, Dietrich K, et al. N-acetylcysteine targets 5 lipoxygenase-derived, toxic lipids and can synergize with prostaglandin E(2) to inhibit ferroptosis and improve outcomes following hemorrhagic stroke in mice. *Ann Neurol* (2018) **84**: 854-72. doi:10.1002/ana.25356

18. Li Y, Cao Y, Xiao J, Shang J, Tan Q, Ping F, et al. Inhibitor of apoptosis-stimulating protein of p53 inhibits ferroptosis and alleviates intestinal ischemia/reperfusion-induced acute lung injury. *Cell Death Differ* (2020) **27**: 2635-50. doi:10.1038/s41418-020-0528-x

19. Deng F, Zhao BC, Yang X, Lin ZB, Sun QS, Wang YF, et al. The gut microbiota metabolite capsiate promotes Gpx4 expression by activating TRPV1 to inhibit intestinal ischemia reperfusion-induced ferroptosis. *Gut Microbes* (2021) **13**: 1-21. doi:10.1080/19490976.2021.1902719

20. Balogh N, Krausz F, Levai P, Ribiczeyne PS, Vajdovich P, Gaal T. Effect of deferoxamine and L-arginine treatment on lipid peroxidation in an intestinal ischaemia-reperfusion model in rats. *Acta Vet Hung* (2002) **50**: 343-56. doi:10.1556/AVet.50.2002.3.10

21. Qiang Z, Dong H, Xia Y, Chai D, Hu R, Jiang H. Nrf2 and STAT3 Alleviates Ferroptosis-Mediated IIR-ALI by Regulating SLC7A11. *Oxid Med Cell Longev* (2020) **2020**: 5146982. doi:10.1155/2020/5146982

22. Qiu YB, Wan BB, Liu G, Wu YX, Chen D, Lu MD, et al. Nrf2 protects against seawater drowning-induced acute lung injury via inhibiting ferroptosis. *Respir Res* (2020) **21**: 232. doi:10.1186/s12931-020-01500-2

23. Fang X, Zhang J, Li Y, Song Y, Yu Y, Cai Z, et al. Malic Enzyme 1 as a Novel Anti-Ferroptotic Regulator in Hepatic Ischemia/Reperfusion Injury. *Adv Sci (Weinh)* (2023) **10**: e2205436. doi:10.1002/advs.202205436

24. Wang C, Yuan W, Hu A, Lin J, Xia Z, Yang CF, et al. Dexmedetomidine alleviated sepsis‑induced myocardial ferroptosis and septic heart injury. *Mol Med Rep* (2020) **22**: 175-84. doi:10.3892/mmr.2020.11114

25. Li N, Wang W, Zhou H, Wu Q, Duan M, Liu C, et al. Ferritinophagy-mediated ferroptosis is involved in sepsis-induced cardiac injury. *Free Radic Biol Med* (2020) **160**: 303-18. doi:10.1016/j.freeradbiomed.2020.08.009

26. Zhang J, Zheng Y, Wang Y, Wang J, Sang A, Song X, et al. YAP1 alleviates sepsis-induced acute lung injury via inhibiting ferritinophagy-mediated ferroptosis. *Front Immunol* (2022) **13**: 884362. doi:10.3389/fimmu.2022.884362

27. Dong H, Qiang Z, Chai D, Peng J, Xia Y, Hu R, et al. Nrf2 inhibits ferroptosis and protects against acute lung injury due to intestinal ischemia reperfusion via regulating SLC7A11 and HO-1. *Aging (Albany Ny)* (2020) **12**: 12943-59. doi:10.18632/aging.103378

28. He R, Liu B, Xiong R, Geng B, Meng H, Lin W, et al. Itaconate inhibits ferroptosis of macrophage via Nrf2 pathways against sepsis-induced acute lung injury. *Cell Death Discov* (2022) **8**: 43. doi:10.1038/s41420-021-00807-3

29. Wang YM, Gong FC, Qi X, Zheng YJ, Zheng XT, Chen Y, et al. Mucin 1 Inhibits Ferroptosis and Sensitizes Vitamin E to Alleviate Sepsis-Induced Acute Lung Injury through GSK3beta/Keap1-Nrf2-GPX4 Pathway. *Oxid Med Cell Longev* (2022) **2022**: 2405943. doi:10.1155/2022/2405943

30. Li J, Li M, Li L, Ma J, Yao C, Yao S. Hydrogen sulfide attenuates ferroptosis and stimulates autophagy by blocking mTOR signaling in sepsis-induced acute lung injury. *Mol Immunol* (2022) **141**: 318-27. doi:10.1016/j.molimm.2021.12.003

31. Wang Y, Chen D, Xie H, Jia M, Sun X, Peng F, et al. AUF1 protects against ferroptosis to alleviate sepsis-induced acute lung injury by regulating NRF2 and ATF3. *Cell Mol Life Sci* (2022) **79**: 228. doi:10.1007/s00018-022-04248-8

32. Shimizu J, Murao A, Nofi C, Wang P, Aziz M. Extracellular CIRP Promotes GPX4-Mediated Ferroptosis in Sepsis. *Front Immunol* (2022) **13**: 903859. doi:10.3389/fimmu.2022.903859

33. Wang J, Zhu Q, Li R, Zhang J, Ye X, Li X. YAP1 protects against septic liver injury via ferroptosis resistance. *Cell Biosci* (2022) **12**: 163. doi:10.1186/s13578-022-00902-7

34. Wei S, Bi J, Yang L, Zhang J, Wan Y, Chen X, et al. Serum irisin levels are decreased in patients with sepsis, and exogenous irisin suppresses ferroptosis in the liver of septic mice. *Clin Transl Med* (2020) **10**: e173. doi:10.1002/ctm2.173

35. Xiao J, Yang Q, Zhang Y, Xu H, Ye Y, Li L, et al. Maresin conjugates in tissue regeneration-1 suppresses ferroptosis in septic acute kidney injury. *Cell Biosci* (2021) **11**: 221. doi:10.1186/s13578-021-00734-x

36. Yao W, Liao H, Pang M, Pan L, Guan Y, Huang X, et al. Inhibition of the NADPH Oxidase Pathway Reduces Ferroptosis during Septic Renal Injury in Diabetic Mice. *Oxid Med Cell Longev* (2022) **2022**: 1193734. doi:10.1155/2022/1193734

37. Elmarakby AA, Ibrahim AS, Katary MA, Elsherbiny NM, El-Shafey M, Abd-Elrazik AM, et al. A dual role of 12/15-lipoxygenase in LPS-induced acute renal inflammation and injury. *Biochim Biophys Acta Mol Cell Biol Lipids* (2019) **1864**: 1669-80. doi:10.1016/j.bbalip.2019.07.009

38. El-Shafei RA, Saleh RM. Pharmacological effects of Vitamin C & E on Diclofenac Sodium intoxicated Rats. *Biomed Pharmacother* (2016) **84**: 314-22. doi:10.1016/j.biopha.2016.09.005

39. Xiao J, Yang Q, Zhang Y, Xu H, Ye Y, Li L, et al. Maresin conjugates in tissue regeneration-1 suppresses ferroptosis in septic acute kidney injury. *Cell Biosci* (2021) **11**: 221. doi:10.1186/s13578-021-00734-x

40. Wang J, Zhu Q, Wang Y, Peng J, Shao L, Li X. Irisin protects against sepsis-associated encephalopathy by suppressing ferroptosis via activation of the Nrf2/GPX4 signal axis. *Free Radic Biol Med* (2022) **187**: 171-84. doi:10.1016/j.freeradbiomed.2022.05.023

41. Dharmalingam P, Talakatta G, Mitra J, Wang H, Derry PJ, Nilewski LG, et al. Pervasive Genomic Damage in Experimental Intracerebral Hemorrhage: Therapeutic Potential of a Mechanistic-Based Carbon Nanoparticle. *Acs Nano* (2020) **14**: 2827-46. doi:10.1021/acsnano.9b05821

42. Zhuo L, Chen X, Sun Y, Wang Y, Shi Y, Bu L, et al. Rapamycin Inhibited Pyroptosis and Reduced the Release of IL-1beta and IL-18 in the Septic Response. *Biomed Res Int* (2020) **2020**: 5960375. doi:10.1155/2020/5960375

43. Liu W, Guo J, Mu J, Tian L, Zhou D. Rapamycin Protects Sepsis-Induced Cognitive Impairment in Mouse Hippocampus by Enhancing Autophagy. *Cell Mol Neurobiol* (2017) **37**: 1195-205. doi:10.1007/s10571-016-0449-x

44. Wang W, Xu R, He P, Xiong Y, Zhao H, Fu X, et al. CircEXOC5 Aggravates Sepsis-Induced Acute Lung Injury by Promoting Ferroptosis Through the IGF2BP2/ATF3 Axis. *J Infect Dis* (2024) **229**: 522-34. doi:10.1093/infdis/jiad337

45. Xu B, Wang H, Chen Z. Puerarin Inhibits Ferroptosis and Inflammation of Lung Injury Caused by Sepsis in LPS Induced Lung Epithelial Cells. *Front Pediatr* (2021) **9**: 706327. doi:10.3389/fped.2021.706327

46. Zhang Y, Zheng L, Deng H, Feng D, Hu S, Zhu L, et al. Electroacupuncture Alleviates LPS-Induced ARDS Through alpha7 Nicotinic Acetylcholine Receptor-Mediated Inhibition of Ferroptosis. *Front Immunol* (2022) **13**: 832432. doi:10.3389/fimmu.2022.832432

47. Li J, Lu K, Sun F, Tan S, Zhang X, Sheng W, et al. Panaxydol attenuates ferroptosis against LPS-induced acute lung injury in mice by Keap1-Nrf2/HO-1 pathway. *J Transl Med* (2021) **19**: 96. doi:10.1186/s12967-021-02745-1

48. Zhang E, Wang J, Chen Q, Wang Z, Li D, Jiang N, et al. Artesunate ameliorates sepsis-induced acute lung injury by activating the mTOR/AKT/PI3K axis. *Gene* (2020) **759**: 144969. doi:10.1016/j.gene.2020.144969

49. Cao TH, Jin SG, Fei DS, Kang K, Jiang L, Lian ZY, et al. Artesunate Protects Against Sepsis-Induced Lung Injury Via Heme Oxygenase-1 Modulation. *Inflammation* (2016) **39**: 651-62. doi:10.1007/s10753-015-0290-2

50. Liu P, Feng Y, Li H, Chen X, Wang G, Xu S, et al. Ferrostatin-1 alleviates lipopolysaccharide-induced acute lung injury via inhibiting ferroptosis. *Cell Mol Biol Lett* (2020) **25**: 10. doi:10.1186/s11658-020-00205-0

51. Yu JB, Shi J, Gong LR, Dong SA, Xu Y, Zhang Y, et al. Role of Nrf2/ARE pathway in protective effect of electroacupuncture against endotoxic shock-induced acute lung injury in rabbits. *Plos One* (2014) **9**: e104924. doi:10.1371/journal.pone.0104924

52. Zhao S, Wang X, Zheng X, Liang X, Wang Z, Zhang J, et al. Iron deficiency exacerbates cisplatin- or rhabdomyolysis-induced acute kidney injury through promoting iron-catalyzed oxidative damage. *Free Radic Biol Med* (2021) **173**: 81-96. doi:10.1016/j.freeradbiomed.2021.07.025

53. Hu Z, Zhang H, Yi B, Yang S, Liu J, Hu J, et al. VDR activation attenuate cisplatin induced AKI by inhibiting ferroptosis. *Cell Death Dis* (2020) **11**: 73. doi:10.1038/s41419-020-2256-z

54. Martin-Sanchez D, Ruiz-Andres O, Poveda J, Carrasco S, Cannata-Ortiz P, Sanchez-Nino MD, et al. Ferroptosis, but Not Necroptosis, Is Important in Nephrotoxic Folic Acid-Induced AKI. *J Am Soc Nephrol* (2017) **28**: 218-29. doi:10.1681/ASN.2015121376

55. Li X, Zou Y, Xing J, Fu YY, Wang KY, Wan PZ, et al. Pretreatment with Roxadustat (FG-4592) Attenuates Folic Acid-Induced Kidney Injury through Antiferroptosis via Akt/GSK-3beta/Nrf2 Pathway. *Oxid Med Cell Longev* (2020) **2020**: 6286984. doi:10.1155/2020/6286984

56. Wang Y, Quan F, Cao Q, Lin Y, Yue C, Bi R, et al. Quercetin alleviates acute kidney injury by inhibiting ferroptosis. *J Adv Res* (2021) **28**: 231-43. doi:10.1016/j.jare.2020.07.007

57. Li D, Liu B, Fan Y, Liu M, Han B, Meng Y, et al. Nuciferine protects against folic acid-induced acute kidney injury by inhibiting ferroptosis. *Br J Pharmacol* (2021) **178**: 1182-99. doi:10.1111/bph.15364

58. Yang L, Guo J, Yu N, Liu Y, Song H, Niu J, et al. Tocilizumab mimotope alleviates kidney injury and fibrosis by inhibiting IL-6 signaling and ferroptosis in UUO model. *Life Sci* (2020) **261**: 118487. doi:10.1016/j.lfs.2020.118487

59. Lo YH, Yang SF, Cheng CC, Hsu KC, Chen YS, Chen YY, et al. Nobiletin Alleviates Ferroptosis-Associated Renal Injury, Inflammation, and Fibrosis in a Unilateral Ureteral Obstruction Mouse Model. *Biomedicines* (2022) **10**. doi:10.3390/biomedicines10030595

60. Li J, Yang J, Zhu B, Fan J, Hu Q, Wang L. Tectorigenin protects against unilateral ureteral obstruction by inhibiting Smad3-mediated ferroptosis and fibrosis. *Phytother Res* (2022) **36**: 475-87. doi:10.1002/ptr.7353

61. Niu B, Lei X, Xu Q, Ju Y, Xu D, Mao L, et al. Protecting mitochondria via inhibiting VDAC1 oligomerization alleviates ferroptosis in acetaminophen-induced acute liver injury. *Cell Biol Toxicol* (2022) **38**: 505-30. doi:10.1007/s10565-021-09624-x

62. Lorincz T, Jemnitz K, Kardon T, Mandl J, Szarka A. Ferroptosis is Involved in Acetaminophen Induced Cell Death. *Pathol Oncol Res* (2015) **21**: 1115-21. doi:10.1007/s12253-015-9946-3

63. Schnellmann JG, Pumford NR, Kusewitt DF, Bucci TJ, Hinson JA. Deferoxamine delays the development of the hepatotoxicity of acetaminophen in mice. *Toxicol Lett* (1999) **106**: 79-88. doi:10.1016/s0378-4274(99)00021-1

64. Park SJ, Cho SS, Kim KM, Yang JH, Kim JH, Jeong EH, et al. Protective effect of sestrin2 against iron overload and ferroptosis-induced liver injury. *Toxicol Appl Pharmacol* (2019) **379**: 114665. doi:10.1016/j.taap.2019.114665

65. Wang Y, Chen Q, Shi C, Jiao F, Gong Z. Mechanism of glycyrrhizin on ferroptosis during acute liver failure by inhibiting oxidative stress. *Mol Med Rep* (2019) **20**: 4081-90. doi:10.3892/mmr.2019.10660

66. Mishima E, Sato E, Ito J, Yamada KI, Suzuki C, Oikawa Y, et al. Drugs Repurposed as Antiferroptosis Agents Suppress Organ Damage, Including AKI, by Functioning as Lipid Peroxyl Radical Scavengers. *J Am Soc Nephrol* (2020) **31**: 280-96. doi:10.1681/ASN.2019060570

67. Alim I, Caulfield JT, Chen Y, Swarup V, Geschwind DH, Ivanova E, et al. Selenium Drives a Transcriptional Adaptive Program to Block Ferroptosis and Treat Stroke. *Cell* (2019) **177**: 1262-79. doi:10.1016/j.cell.2019.03.032

68. Guan X, Li X, Yang X, Yan J, Shi P, Ba L, et al. The neuroprotective effects of carvacrol on ischemia/reperfusion-induced hippocampal neuronal impairment by ferroptosis mitigation. *Life Sci* (2019) **235**: 116795. doi:10.1016/j.lfs.2019.116795

69. Cui L, Zhang X, Yang R, Liu L, Wang L, Li M, et al. Baicalein is neuroprotective in rat MCAO model: role of 12/15-lipoxygenase, mitogen-activated protein kinase and cytosolic phospholipase A2. *Pharmacol Biochem Behav* (2010) **96**: 469-75. doi:10.1016/j.pbb.2010.07.007

70. Yang W, Liu X, Song C, Ji S, Yang J, Liu Y, et al. Structure-activity relationship studies of phenothiazine derivatives as a new class of ferroptosis inhibitors together with the therapeutic effect in an ischemic stroke model. *Eur J Med Chem* (2021) **209**: 112842. doi:10.1016/j.ejmech.2020.112842

71. Chen X, Gao C, Yan Y, Cheng Z, Chen G, Rui T, et al. Ruxolitinib exerts neuroprotection via repressing ferroptosis in a mouse model of traumatic brain injury. *Exp Neurol* (2021) **342**: 113762. doi:10.1016/j.expneurol.2021.113762

72. Liang H, Tang T, Huang H, Li T, Gao C, Han Y, et al. Peroxisome proliferator-activated receptor-gamma ameliorates neuronal ferroptosis after traumatic brain injury in mice by inhibiting cyclooxygenase-2. *Exp Neurol* (2022) **354**: 114100. doi:10.1016/j.expneurol.2022.114100

73. Xie B, Wang Y, Lin Y, Mao Q, Feng J, Gao G, et al. Inhibition of ferroptosis attenuates tissue damage and improves long-term outcomes after traumatic brain injury in mice. *Cns Neurosci Ther* (2019) **25**: 465-75. doi:10.1111/cns.13069

74. Huang L, He S, Cai Q, Li F, Wang S, Tao K, et al. Polydatin alleviates traumatic brain injury: Role of inhibiting ferroptosis. *Biochem Biophys Res Commun* (2021) **556**: 149-55. doi:10.1016/j.bbrc.2021.03.108

75. Liu H, He S, Wang J, Li C, Liao Y, Zou Q, et al. Tetrandrine Ameliorates Traumatic Brain Injury by Regulating Autophagy to Reduce Ferroptosis. *Neurochem Res* (2022) **47**: 1574-87. doi:10.1007/s11064-022-03553-9

76. Wu C, Du M, Yu R, Cheng Y, Wu B, Fu J, et al. A novel mechanism linking ferroptosis and endoplasmic reticulum stress via the circPtpn14/miR-351-5p/5-LOX signaling in melatonin-mediated treatment of traumatic brain injury. *Free Radic Biol Med* (2022) **178**: 271-94. doi:10.1016/j.freeradbiomed.2021.12.007
